# Treatment-induced arteriolar revascularization and miR-126 enhancement in bone marrow niche protect leukemic stem cells in AML

**DOI:** 10.1186/s13045-021-01133-y

**Published:** 2021-08-09

**Authors:** Bin Zhang, Le Xuan Truong Nguyen, Dandan Zhao, David E. Frankhouser, Huafeng Wang, Dinh Hoa Hoang, Junjing Qiao, Christina Abundis, Matthew Brehove, Yu-Lin Su, Yuxin Feng, Anthony Stein, Lucy Ghoda, Adrianne Dorrance, Danilo Perrotti, Zhen Chen, Anjia Han, Flavia Pichiorri, Jie Jin, Tijana Jovanovic-Talisman, Michael A. Caligiuri, Calvin J. Kuo, Akihiko Yoshimura, Ling Li, Russell C. Rockne, Marcin Kortylewski, Yi Zheng, Nadia Carlesso, Ya-Huei Kuo, Guido Marcucci

**Affiliations:** 1 Department of Hematological Malignancies Translational Science, Gehr Family Center for Leukemia Research, City of Hope Medical Center and Beckman Research Institute, 1500 E Duarte Road, Duarte, CA 91010 USA; 2 Department of Population Science, City of Hope, Duarte, CA USA; 3 Department of Hematology, The First Affiliated Hospital, College of Medicine, Zhejiang University, Hangzhou, Zhejiang People’s Republic of China; 4 Department of Pathology, The First Affiliated Hospital, Sun Yat-Sen University, Guangzhou, Guangdong People’s Republic of China; 5 Department of Molecular Medicine, City of Hope, Duarte, CA USA; 6 Department of Immuno-Oncology, City of Hope, Duarte, CA USA; 7 Division of Experimental Hematology and Cancer Biology, Children’s Hospital Medical Center, Cincinnati, OH USA; 8 The Ohio State University, Columbus, OH USA; 9 University of Maryland, Baltimore, MD USA; 10 Department of Diabetes Complications and Metabolism, City of Hope, Duarte, CA USA; 11 Department of Medicine, Division of Hematology, Stanford University, Stanford, CA USA; 12 Department of Microbiology and Immunology, Keio University School of Medicine, Tokyo, Japan; 13 Division of Mathematical Oncology, Department of Computational and Quantitative Medicine, Beckman Research Institute, City of Hope Medical Center, Duarte, CA USA

**Keywords:** Acute myeloid leukemia, BM vascular niche, TNFα, miR-126, Leukemic stem cell, Treatment resistance

## Abstract

**Background:**

During acute myeloid leukemia (AML) growth, the bone marrow (BM) niche acquires significant vascular changes that can be offset by therapeutic blast cytoreduction. The molecular mechanisms of this vascular plasticity remain to be fully elucidated. Herein, we report on the changes that occur in the vascular compartment of the FLT3-ITD+ AML BM niche pre and post treatment and their impact on leukemic stem cells (LSCs).

**Methods:**

BM vasculature was evaluated in FLT3-ITD+ AML models (*Mll*^PTD/WT^/*Flt3*^ITD/ITD^ mouse and patient-derived xenograft) by 3D confocal imaging of long bones, calvarium vascular permeability assays, and flow cytometry analysis. Cytokine levels were measured by Luminex assay and miR-126 levels evaluated by Q-RT-PCR and miRNA staining. Wild-type (wt) and *Mll*^PTD/WT^/*Flt3*^ITD/ITD^ mice with endothelial cell (EC) miR-126 knockout or overexpression served as controls. The impact of treatment-induced BM vascular changes on LSC activity was evaluated by secondary transplantation of BM cells after administration of tyrosine kinase inhibitors (TKIs) to *Mll*^PTD/WT^/*Flt3*^ITD/ITD^ mice with/without either EC miR-126 KO or co-treatment with tumor necrosis factor alpha (TNFα) or anti-miR-126 miRisten.

**Results:**

In the normal BM niche, CD31^+^Sca-1^high^ ECs lining arterioles have miR-126 levels higher than CD31^+^Sca-1^low^ ECs lining sinusoids. We noted that during FLT3-ITD+ AML growth, the BM niche lost arterioles and gained sinusoids. These changes were mediated by TNFα, a cytokine produced by AML blasts, which induced EC miR-126 downregulation and caused depletion of CD31^+^Sca-1^high^ ECs and gain in CD31^+^Sca-1^low^ ECs. Loss of miR-126^high^ ECs led to a decreased EC miR-126 supply to LSCs, which then entered the cell cycle and promoted leukemia growth. Accordingly, antileukemic treatment with TKI decreased the BM blast-produced TNFα and increased miR-126^high^ ECs and the EC miR-126 supply to LSCs. High miR-126 levels safeguarded LSCs, as shown by more severe disease in secondary transplanted mice. Conversely, EC miR-126 deprivation via genetic or pharmacological EC miR-126 knock-down prevented treatment-induced BM miR-126^high^ EC expansion and in turn LSC protection.

**Conclusions:**

Treatment-induced CD31^+^Sca-1^high^ EC re-vascularization of the leukemic BM niche may represent a LSC extrinsic mechanism of treatment resistance that can be overcome with therapeutic EC miR-126 deprivation.

**Graphic abstract:**

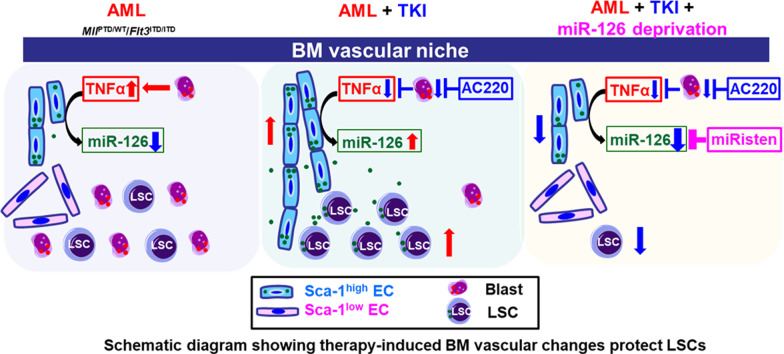

**Supplementary Information:**

The online version contains supplementary material available at 10.1186/s13045-021-01133-y.

## Background

Acute myeloid leukemia (AML) is a hematopoietic malignancy characterized by somatic mutations occurring in the hematopoietic stem cells (HSCs) and progenitor cells that block hematopoietic differentiation and promote accumulation of leukemic “blasts” in the bone marrow (BM) and/or other extramedullary organs [1]. To date, despite a deep molecular understanding of the pathogenesis, the development of molecular targeting therapeutics and the broadened use of allogeneic HSC transplantation, the overall outcome of AML patients remains poor. Disease refractoriness to initial therapy or post-remission disease relapse [2] are likely due to persistence of treatment-resistant leukemic stem cells (LSCs) [3]. These are primitive leukemic cells capable of unlimited self-renewal and disease initiation [4, 5] and reside in a leukemic BM niche that also comprises several types of non-hematopoietic cells and that preferentially supports homeostasis and competitive growth of LSCs over those of HSCs [6, 7].

Mechanisms of treatment resistance in cancer are multifaceted and often result from the acquisition of genetic mutations that enable malignant cells to escape the therapeutic pressure. Recently, other non-genetic mechanisms of treatment resistance have been also described [8]. While these reported mechanisms have been mainly reported as intrinsic to malignant cells, it is possible that they also include mechanisms that are extrinsic to malignant cells, such as those involving the microenvironment and that protect malignant cells during treatment exposure [7].

Utilizing the FMS-like tyrosine kinase 3 *(FLT3)* gene internal tandem duplication (ITD) (FLT3-ITD) knock-in mouse and FLT3-ITD+ AML patient-derived xenograft (PDX) models that recapitulate features of human FLT3-ITD+ AML, we report here on previously unrecognized non-genetic, extrinsic mechanisms of treatment resistance in LSCs that involve the vascular compartment of the leukemic BM niche and that are mediated by a TNFα-miR-126 axis in the BM endothelial cells (ECs). *FLT3-*ITD occurs in approximately 25% of AML patients and the mutated gene encodes a mutant receptor with aberrant, ligand-independent tyrosine kinase (TK) activity that confers growth and survival advantages to leukemic blasts [9]. FLT3-ITD+ AML patients are treated with TK inhibitors (TKIs) in combination with chemotherapy. Although the addition of TKIs to chemotherapy confers a clinical advantage compared to chemotherapy alone, it is not curative in the majority of cases, suggesting treatment resistance arising over time [10].

## Methods

An extended description of the methods is in the Additional file 1.

### Human samples

Normal peripheral blood (PB) and BM samples were obtained from healthy donors at the City of Hope National Medical Center (COHNMC). AML samples were obtained from patients from the COHNMC (Additional file 1: Table S1). Mononuclear cells (MNCs) were isolated using Ficoll separation. When necessary, CD34^+^ cells were then isolated using a positive magnetic bead selection protocol (Miltenyi Biotech, Germany). Sample acquisition was approved by the Institutional Review Boards at the COHNMC, in accordance with an assurance filed with and approved by the Department of Health and Human Services and met all requirements of the Declaration of Helsinki. Healthy donors and AML patients were consented on the IRB # 06229 and IRB# 18067 protocols, respectively.

### Animal studies

The *Mll*^PTD/WT^/*Flt3*^ITD/ITD^ mouse, an AML model in C57Bl/6J (B6) background (CD45.2), was generated and genotyped as previously described [11]. The *Mll*^PTD/WT^/*Flt3*^ITD/ITD^ mice (CD45.2, B6) were also bred with CD45.1 B6 mice to produce CD45.1/CD45.2 *Mll*^PTD/WT^/*Flt3*^ITD/ITD^ mice as donors for transplant experiments. To obtain conditional miR-126 knock-out (KO) or Spred1 KO in ECs, we bred miR-126^flox/flox^ (CD45.2 B6) [12] and Spred1^flox/flox^ (CD45.2 B6) [13] mice with Tie2-cre (CD45.2 B6, from The Jackson Laboratory, 008863) mice and obtained *miR-126*^*flox/flox*^*Tie2-cre*+ (EC miR-126 KO, also called *miR-126*^*ECΔ/Δ*^) and *Spred1*^*flox/flox*^*Tie2-cre*+ (EC Spred1 KO, also called *Spred1*^*ECΔ/Δ*^, representing a functional EC miR-126 overexpressing model) mice. *Tie2-CreER/TdTomato/Tg(Ly6a-GFP)* double fluorescent EC/Sca-1 reporter mice were generated by crossing *Tie2-CreER/TdTomato* mice (CD45.2 B6), in which BM ECs bearing the Tie2-promoter driven TdTomato color upon tamoxifen administration (EC-tdTomato^+^, EC reporter mice) [14, 15], with *Tg(Ly6a-EGFP)* (Sca-1-GFP^+^, Sca-1 reporter mice, from The Jackson Laboratory, 012643), which allowed us to visualize Sca-1^high^ EC (tdTomato^+^GFP^high^) and Sca-1^low^ EC (tdTomato^+^GFP^low^) lined vessels based on the combined expression of these two endogenous reporters. To obtain conditional EC miR-126 KO reporter mice, we also bred *Tie2-CreER/TdTomato/Tg(Ly6a-GFP)* double fluorescent reporter mice with *miR-126*^*flox/flox*^ mice and obtained inducible EC miR-126 KO reporter mice [i.e., *miR-126*^*flox/flox*^*/Tie2-CreER/TdTomato/Tg(Ly6a-GFP)*, miR-126 KO in ECs upon tamoxifen administration].

To evaluate the impact of EC miR-126 on leukemia-induced vascular changes and in turn on LSC burden, BM MNCs from AML (i.e., *Mll*^PTD/WT^/*Flt3*^ITD/ITD^, CD45.1/CD45.2 B6) or from normal wild-type (wt, CD45.1 B6) mice were transplanted into Cre+ or Cre- *miR-126*^*flox/flox*^*/Tie2-cre*, *Spred1*^*flox/flox*^*/Tie2-cre*, and tamoxifen-treated *miR-126*^*flox/flox*^*/Tie2-CreER/TdTomato/Tg(Ly6a-GFP)* reporter mice (all CD45.2 B6). To study the vascular changes induced by human FLT3-ITD+ AML blast growth, NOD.Cg-Prkdc^scid^ Il2rg^tm1Wjl^ Tg(CMV-IL3, CSF2, KITLG)1Eav/MloySzJ mice (NSG-SGM3 or NSGS, from The Jackson Laboratory, 013062) were transplanted with normal cord blood (CB) CD34^+^ cells or with human FLT3-ITD+ AML blasts (patient-derived xenograft, PDX).

### Immunofluorescent staining and 3D confocal imaging of long bones

Long bones (femurs and/or tibias) from the mice were processed, sectioned and imaged as described previously [16] with ad-hoc modifications (see Additional file 1 for details).

### Intravital imaging

Intravital confocal microscopy was used to image the calvarium BM vasculature to study the vascular permeability, as previously described [17] (see Additional file 1 for details).

### Statistical analysis

Comparison between groups was performed by two-tailed, paired or unpaired Student's *t*-test, adjusting for multiple comparisons as appropriate. The log-rank test was used to assess significant differences between survival curves. All statistical analyses were performed using Prism version 8.0 software (GraphPad Software). Sample sizes chosen are indicated in the individual figure legends and were not based on formal power calculations to detect prespecified effect sizes but were based on previous experience with similar models. All of the in vitro experiments were performed 3–6 times using biologically independent samples; the in vivo experiments were performed using 6–16 mice in each group. *p* values < 0.05 were considered significant. Results shown represent mean ± SEM. ∗*p* ≤ 0.05, ∗∗*p* < 0.01, ∗∗∗*p* < 0.001, ∗∗∗∗*p* < 0.0001.

## Results

### Bone marrow vasculature of normal and leukemic mice

To determine how the vascular compartment of the BM niche adapts to leukemia growth and subsequently to antileukemic treatments, we performed immunofluorescence staining and 3D confocal imaging of vessels in the long bones of normal versus (vs) leukemic mice. To this end, we first analyzed normal mice and identified CD31^+^α-SMA^+^Sca-1^high^Endomucin(Emcn)^−^ vessels as arteries (Additional file 1: Fig. S1a–c, blue arrow), CD31^+^α-SMA^−^Sca-1^high^Emcn^−^ vessels as arterioles (Additional file 1: Fig. S1a–e, yellow arrow), and CD31^+^α-SMA^−^Sca-1^low^Emcn^+^ vessels as sinusoids (Additional file 1: Fig. S1a–e, white arrow) [18–21]. In a double fluorescent reporter mouse [i.e., tamoxifen-treated Tie2-CreER/TdTomato/Tg(Ly6a-GFP)], Sca-1^high^ EC (i.e., tdTomato^+^GFP^high^) and Sca-1^low^ EC (i.e., tdTomato^+^GFP^low^) lined vessels were also morphologically consistent respectively with arteries and arterioles (both are tdTomato^+^GFP^high^ but with different size; Additional file 1: Fig. S1f, blue arrow indicates artery and yellow arrows indicate arteriole) and sinusoids (Additional file 1: Fig. S1f, white arrow). Since Sca-1^high^ expression appeared to be restricted to arteries and arterioles, we then utilized a simplified CD31^+^Sca-1^high^ and CD31^+^Sca-1^low^ staining along with morphology examination to mark respectively arterioles and sinusoids in the BM niche [19, 22].

To study changes of the vascular compartment of the BM niche during leukemia growth, we then utilized the *Mll*^PTD/WT^/*Flt3*^ITD/ITD^ mouse, a model that recapitulates phenotypic, cytogenetic, molecular and pathological features of human FLT3-ITD+ AML, a relatively frequent molecular subset of the disease [11]. Of note, we will refer hereafter to “wt” or “*Mll*^PTD/WT^/*Flt3*^ITD/ITD^” to indicate the mouse genotype and to “normal” or “leukemic” to indicate the disease status.

**Fig. 1:**
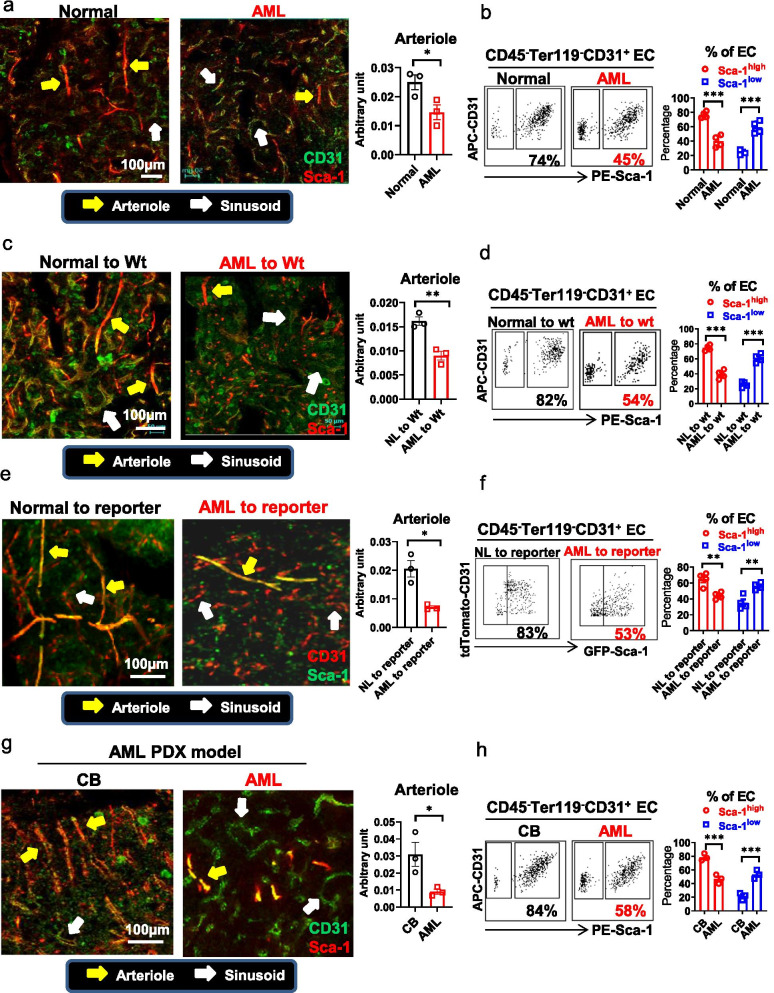
Vascular remodeling of the leukemic BM niche. **a** CD31 (FITC) and Sca-1 (PE) immunofluorescence (IF) staining (left) and quantification (right) of CD31^+^Sca-1^high^ EC-lined vessels (i.e., arterioles) in the femurs from normal wt and AML mouse (*n* = 3 mice per group). **b** Representative plots (left) and aggregate results (right) of BM EC Sca-1^high^ and Sca-1^low^ subfractions from normal wt and AML mouse by flow cytometry analysis (*n* = 4 mice per group). **c** CD31 (FITC) and Sca-1 (PE) IF staining (left) and quantification (right) of CD31^+^Sca-1^high^ EC-lined vessels (i.e., arterioles) in the tibias from wt recipient mice receiving BM mononuclear cells (MNCs) from normal mice (Normal or NL to wt, 1 × 10^6^/mouse, *n* = 4 mice) or from diseased *Mll*^PTD/WT^/*Flt3*^ITD/ITD^ AML mice (AML to wt, 1 × 10^6^/mouse, *n* = 4 mice). **d** Representative plots (left) and aggregate results (right) of BM EC Sca-1^high^ and Sca-1^low^ subfractions from the same mice as in (**c**). **e** Confocal imaging of EC-tdTomato and Sca-1-GFP (left) and quantification (right) of Sca-1-GFP^high^ EC-lined vessels (i.e., arterioles) in the tibias from tamoxifen-treated Tie2-CreER/TdTomato/Tg(Ly6a-GFP) recipient mice receiving BM MNCs from normal wt mice (Normal or NL to reporter, 1 × 10^6^/mouse, *n* = 4 mice) or from leukemic *Mll*^PTD/WT^/*Flt3*^ITD/ITD^ mice (AML to reporter, 1 × 10^6^/mouse, *n* = 4 mice). BM engraftment rates were more than 70% when the mice were analyzed. **f** Representative plots (left) and aggregate results (right) of BM Sca-1-GFP^high^ and Sca-1-GFP^low^ subfractions in BM ECs (CD45^−^Ter119^−^tdTomato^+^) from the same mice as in (**e**). **g** CD31 (FITC) and Sca-1 (PE) IF staining (left) and quantification (right) of CD31^+^Sca-1^high^ EC-lined vessels (i.e., arterioles) in the tibias from NSGS mice receiving CB CD34^+^ cells (1 × 10^5^/mouse, *n* = 3 mice) or receiving human FLT3-ITD+ AML blasts (2 × 10^6^/mouse, *n* = 3 mice). BM engraftment rates were more than 70% when the mice were analyzed. **h** Representative plots (left) and aggregate results (right) of BM EC Sca-1^high^ and Sca-1^low^ subfractions from the same mice as in (**g**). For **a**, **c**, and **g**: yellow arrows indicate CD31^+^Sca-1^high^ EC-lined vessels; white arrows indicate CD31^+^Sca-1^low^ EC-lined vessels; for **e**: yellow arrows indicate Sca-1^high^ EC-lined vessels (i.e., tdTomato^+^GFP^high^); white arrows indicate Sca-1^low^ EC-lined vessels (i.e., tdTomato^+^GFP^low^); scale bars represent a size of 100 µm. Results represent mean ± SEM. Significance values: *p < 0.05; **p < 0.01; ***p < 0.001; *ns* not significant

Firstly, we noticed a significant decrease in CD31^+^Sca-1^high^ EC lined vessels (i.e., arterioles) in the leukemic *Mll*^PTD/WT^/*Flt3*^ITD/ITD^ mice compared with normal wt mice (Fig. 1a; Additional file 1: Fig. S2a). These results were corroborated by a flow cytometry analysis showing an overall increase in total BM ECs (CD45^−^Ter119^−^CD31^+^) in the leukemic mice (Additional file 1: Fig. S2b, c) but with a lower frequency of CD31^+^Sca-1^high^ ECs and a higher frequency of CD31^+^Sca-1^low^ ECs compared with normal wt mice (Fig. 1b; Additional file 1: Fig. S2d). Similar results were also obtained when normal wt mice were engrafted with BM MNCs from congenic leukemic *Mll*^PTD/WT^/*Flt3*^ITD/ITD^ donors and compared with controls engrafted with BM MNCs from congenic normal wt donors (Fig. 1c, d; Additional file 1: Fig. S2e).

To validate these findings, we then transplanted BM MNCs from leukemic *Mll*^PTD/WT^/*Flt3*^ITD/ITD^ mice or from normal wt mice into the tamoxifen-induced Tie2-CreER/TdTomato/Tg(Ly6a-GFP) double reporter mice. At 4 weeks after transplantation, we observed reduced Sca-1^high^ EC-lined vessels (i.e., tdTomato^+^ GFP^high^) in the recipients of AML BM MNCs compared with recipients of normal BM MNCs (Fig. 1e; Additional file 1: Fig. S2f). Consistent with these results, flow cytometry showed reduced Sca-1^high^ ECs (i.e., tdTomato^+^ GFP^high^) and increased Sca-1^low^ ECs (i.e., tdTomato^+^ GFP^low^) in the double reporter mice engrafted with AML BM MNCs compared with those engrafted with normal BM MNCs (Fig. 1f; Additional file 1: Fig. S2g).

Next, to assess the relevance of these changes to the human disease, we transplanted human primary FLT3-ITD+ AML blasts into NSG-SGM3 (NSGS) mice and generated a patient-derived xenograft (PDX) model. Similar to the murine AML models, the FLT3-ITD+ PDX showed fewer BM CD31^+^Sca1^high^ ECs and arterioles as compared with NSGS mice engrafted with normal CB CD34^+^ cells (Fig. 1g, h; Additional file 1: Fig. S3).

In the BM niche, CD31^+^Sca-1^high^ ECs reportedly line impermeable vessels such as arteries and arterioles, and CD31^+^Sca-1^low^ ECs border permeable vessels such as sinusoids [21]. To this end, we imaged the calvarium of normal Sca-1 reporter [i.e., Tg(Ly6a-GFP)] mice with intravital confocal microscopy. Prior to imaging, mice were administered TRITC-dextran (150 kDa, red) intravenously to identify the vasculature [23, 24]. As expected, dextran leakage was visible as diffuse staining preferentially around Sca-1-GFP^low^ vessels, rather than Sca-1-GFP^high^ vessels (Additional file 1: Fig. S4). Consistent with a decrease in CD31^+^Sca-1^high^ vessels (i.e., arterioles) (Fig. 1a, g), we observed increased vessel permeability in the *Mll*^PTD/WT^/*Flt3*^ITD/ITD^ AML mouse (TRITC-150 kDa dextran, red) and FLT3-ITD+ PDX (FITC-150 kDa dextran, green) (Additional file 1: Fig. S5a, b). Similar results were obtained when we transplanted BM MNCs from leukemic *Mll*^PTD/WT^/*Flt3*^ITD/ITD^ mice or from normal wt mice into wt (FITC-150 kDa dextran, green; Additional file 1: Fig. S5c), EC reporter (i.e., tamoxifen-treated Tie2-CreER/TdTomato; FITC-150 kDa dextran, green; Additional file 1: Fig. S5d), or EC/Sca-1 double reporter [i.e., tamoxifen-treated Tie2-CreER/TdTomato/Tg(Ly6a-EGFP); Alex647-150 kDa dextran, blue; Additional file 1: Fig. S5e] recipient mice.

Thus, using different AML models and imaging techniques, flow cytometric analyses and permeability studies of the BM niche, we showed that FLT3-ITD+ AML growth led to a decrease of CD31^+^Sca-1^high^ vessels (i.e., arterioles) in the BM niche.

### TNFα mediates loss of CD31^+^Sca-1^high^ vessels in the leukemic BM niche

**Fig. 2:**
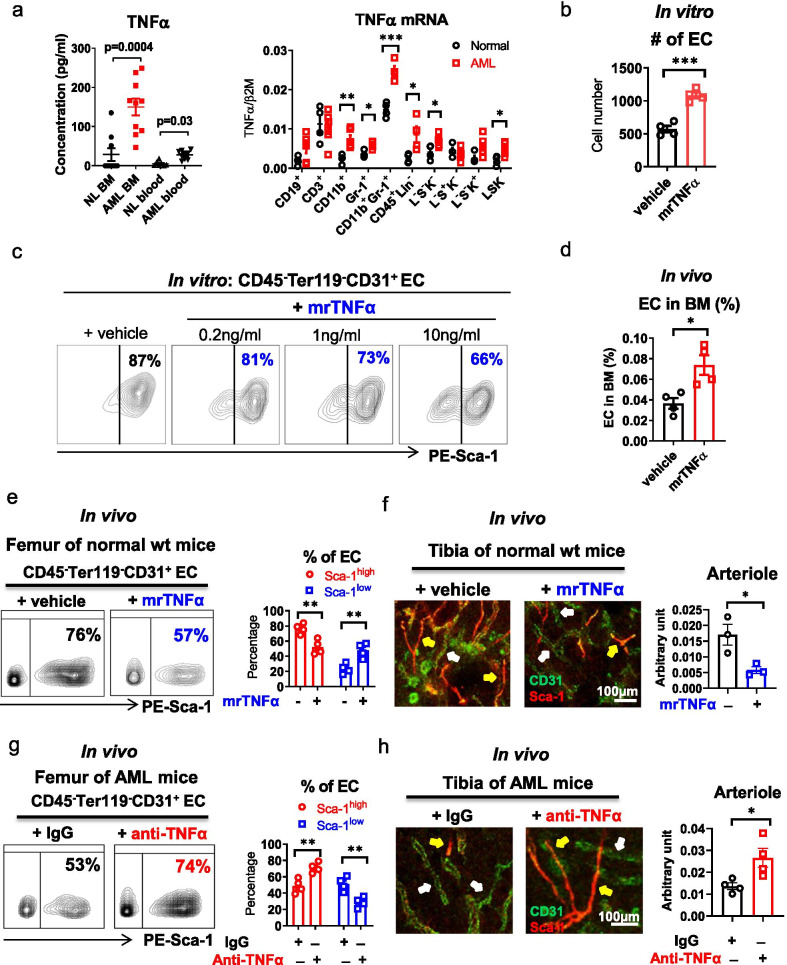
TNFα mediates loss of CD31^+^Sca-1^high^ vessels in the BM niche of FLT3-ITD+ AML. **a** Levels of TNFα (pg/ml, left) in the BM and blood analyzed by Luminex assay (*n* = 10 mice per group) and levels of TNFα mRNA (right) in the BM CD45^+^ cell subpopulations analyzed by Q-RT-PCR (*n* = 4–7 mice for each population) from wt and *Mll*^PTD/WT^/*Flt3*^ITD/ITD^ AML mice. **b** and **c** Number of ECs from normal wt mice after in vitro exposure to murine recombinant (mr) TNFα (1 ng/ml) or vehicle for 96 h (**b**) and frequency of Sca-1^high^ EC subfraction after in vitro exposure to mrTNFα (0, 0.2, 1 and 10 ng/ml) for 96 h (**c**), analyzed by flow cytometry. One of the three independent experiments with similar results is shown. **d**–**f** Normal wt mice treated with vehicle or mrTNFα (1 µg/day, ip, 3 weeks) were evaluated by flow cytometry (*n* = 4 mice per group) for frequencies of BM ECs (**d**) and Sca-1^high^ and Sca-1^low^ EC subfractions (**e**, left, representative plots; right, aggregate results) and by tibial CD31 (FITC) and Sca-1 (PE) IF staining (**f**, left) and quantification (**f**, right; *n* = 3 mice per group) of CD31^+^Sca1^high^ EC-lined vessels. For **f**: yellow arrows indicate CD31^+^Sca-1^high^ EC-lined vessels; white arrows indicate CD31^+^Sca-1^low^ EC-lined vessels. Scale bars represent a size of 100 µm. **g** and **h** Diseased *Mll*^PTD/WT^/*Flt3*^ITD/ITD^ AML mice treated with IgG or anti-TNFα Abs (1 mg/day, ip, 4 times/week, 3 weeks; *n* = 4 mice per group) were evaluated by flow cytometry for frequencies of BM Sca-1^high^ and Sca-1^low^ EC subfractions (**g**, left, representative plots; right, aggregate results) and by tibial CD31 (FITC) and Sca-1 (PE) IF staining (**h** left) and quantification (**h** right) of CD31^+^Sca1^high^ EC-lined vessels. For **h:** yellow arrows indicate CD31^+^Sca-1^high^ EC-lined vessels; white arrows indicate CD31^+^Sca-1^low^ EC-lined vessels. Scale bars represent a size of 100 µm. Results represent mean ± SEM. Significance values: *p < 0.05; **p < 0.01; ***p < 0.001; *ns* not significant

To gain insights into the mechanisms leading to CD31^+^Sca-1^high^ EC and arteriole depletion in the BM niche during leukemia growth, we hypothesized that these effects could be associated with certain secretory features of the proliferating leukemic blasts. We therefore measured levels of cytokines and chemokines in the blood and BM of age- and gender-matched leukemic *Mll*^PTD/WT^/*Flt3*^ITD/ITD^ and normal wt mice. TNFα was the only cytokine significantly elevated in the BM of leukemic mice (Fig. 2a, left; Additional file 1: Fig. S6a–m). Higher TNFα mRNA levels were observed in myeloid cells and CD45^+^Lin^−^ progenitors [i.e., Lin^−^Sca-1^−^c-Kit^−^ (L^−^S^−^K^−^) and Lin^−^Sca-1^+^c-Kit^+^ (LSK)] from the leukemic *Mll*^PTD/WT^/*Flt3*^ITD/ITD^ mice compared to the counterparts from normal wt mice (Fig. 2a, right), suggesting an overproduction of this cytokine by the clonal myeloid subpopulations in the leukemic mice. In vitro treatment of BM ECs from normal wt mice with murine recombinant (mr) TNFα (mrTNFα: 1 ng/ml) for 96 h (h) recapitulated the observations in the leukemic mice, with an expansion of ECs (Fig. 2b), a decrease of CD31^+^Sca-1^high^ EC subfraction and an increase of CD31^+^Sca-1^low^ EC subfraction (Fig. 2c; Additional file 1: Fig. S7a). Both TNFα receptor type 1 and 2 (TNFαR1 and TNFαR2) were found to be co-expressed on the surface of CD31^+^Sca-1^high^ ECs (Additional file 1: Fig. S7b) and TNFαR1 and TNFαR2 blocking antibodies (Abs) reversed the mrTNFα effect (Additional file 1: Fig. S7c).

Consistent with these results, normal wt mice given mrTNFα [1 µg/day, intraperitoneal (ip) injection, 3 weeks] [25, 26] showed a significant increase of BM ECs (Fig. 2d), and loss of CD31^+^Sca-1^high^ ECs and arterioles compared with vehicle-treated controls (Fig. 2e, f). Accordingly, normal wt mice engrafted with BM from leukemic *Mll*^PTD/WT^/*Flt3*^ITD/ITD^ donors had higher levels of BM TNFα (Additional file 1: Fig. S7d) and loss of CD31^+^Sca-1^high^ ECs and arterioles (Fig. 1c, d; Additional file 1: Fig. S2e), compared with mice engrafted with BM from normal wt donors. Similar results were obtained in NSGS mice engrafted with human FLT3-ITD+ AML blasts compared with NSGS mice engrafted with normal CB CD34^+^ cells (Fig. 1g, h; Additional file 1: Fig. S3, S7e, f). In vivo treatment of *Mll*^PTD/WT^/*Flt3*^ITD/ITD^ AML mice with TNFα-neutralizing Ab (anti-TNFα; MP6-XT22, Biolegend; 1 mg/day, ip, 4 times/week for 3 weeks) [27] rescued the loss of CD31^+^Sca-1^high^ ECs and arterioles (Fig. 2g, h) otherwise observed in the leukemic BM niche.

Taken altogether, these results support a TNFα-induced depletion of CD31^+^Sca-1^high^ vessels in the leukemic BM niche during FLT3-ITD+ AML growth.

### TNFα depletes CD31^+^Sca-1^high^ ECs via miR-126 downregulation

The vascular changes induced by TNFα in BM niche were strikingly similar to what we observed in the mice with miR-126 KO in ECs (*miR-126*^*flox/flox*^*Tie2-cre*+, hereafter called *miR-126*^*ECΔ/Δ*^) obtained by crossing *miR-126*^*flox/flox*^ with Tie2-cre mice. This led us to hypothesize that TNFα induces vascular changes during leukemia growth partly via miR-126 downregulation. To this end, it has been reported that in mature ECs, miR-126 contributes to the maintenance of vascular integrity and inhibition of endothelial permeability, which are main features of arterial and arteriolar vessels [12, 28, 29]. In the normal mouse BM niche, we found that ECs expressed at least a log-fold higher level of miR-126 than normal long-term (LT) HSCs (LSK Fit3^−^CD150^+^CD48^−^) and other non-hematopoietic stromal cells, including osteoblasts (OBs; CD45^−^Ter119^−^CD31^−^CD166^+^Sca-1^−^) and mesenchymal stromal progenitors (MSPs; CD45^−^Ter119^−^CD31^−^CD166^−^Sca-1^+^) [30] (Additional file 1: Fig. S8a, b). Within the ECs, Sca-1^high^ ECs expressed significantly higher levels of miR-126 (Additional file 1: Fig. S8c, d) and lower levels of miR-126 verified targets (i.e., *Vcam1* and *Spred1)* [12, 28, 29] (Additional file 1: Fig. S8e) than Sca-1^low^ ECs. In addition, transcriptomes of Sca-1^high^ and Sca-1^low^ ECs from normal wt mice showed different gene expression profiles (Additional file 1: Fig. S8f), with pathways regulating angiogenesis, cell migration and vasculature development being activated in Sca-1^high^ ECs (Additional file 1: Table S2). Q-RT-PCR assays (Additional file 1: Table S3) validated these findings (Additional file 1: Fig. S8g, right panel).

**Fig. 3:**
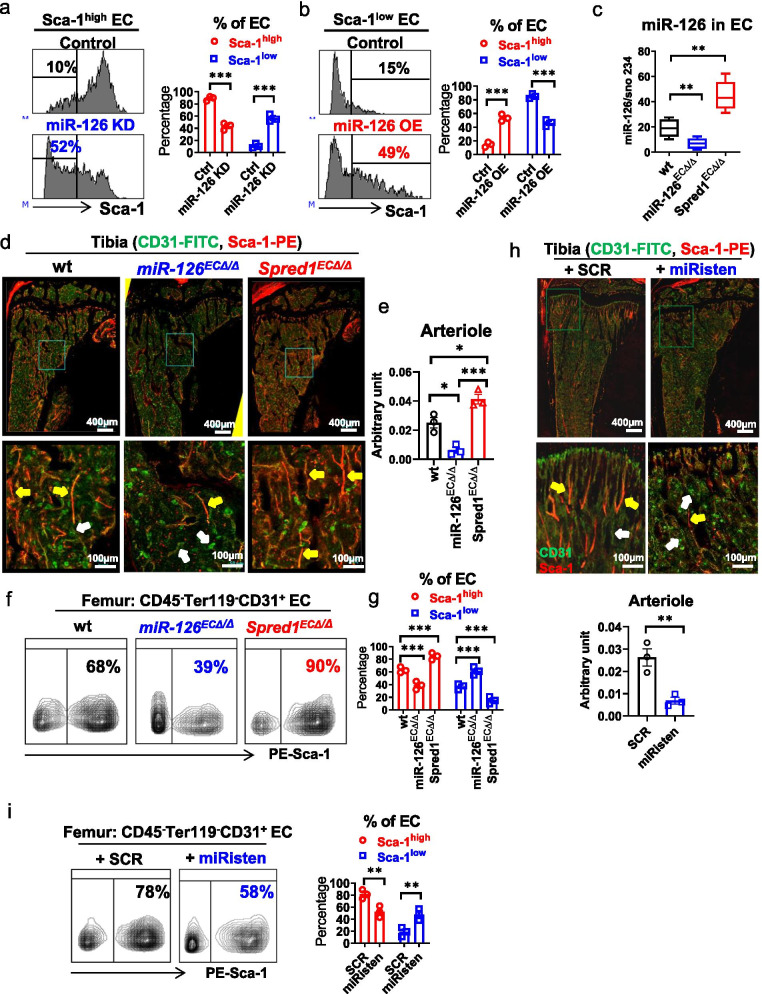
EC miR-126 downregulation associates with CD31^+^Sca-1^high^ EC depletion in the BM niche. **a** Representative plots (left) and aggregate results (right) of Sca-1^low^ frequency in cultured Sca-1^high^ ECs upon miR-126 knock-down (KD) by GFP-expressing miRZip anti-miR-126 lentivirus transduction, as analyzed by flow cytometry (*n* = 3). **b** Representative plots (left) and aggregate results (right) of Sca-1^high^ frequency in cultured Sca-1^low^ ECs upon miR-126 over-expression (OE) by GFP-expressing miR-126 precursor lentivirus transduction, as analyzed by flow cytometry (*n* = 3). **c** miR-126 levels in ECs from the BM of wt, *miR-126*^*ECΔ/Δ*^ (EC miR-126 KO) and *Spred1*^*ECΔ/Δ*^ (EC miR-126 OE) mice by Q-RT-PCR (*n* = 4 mice per group). **d** and **e** CD31 (FITC) and Sca-1 (PE) IF staining (**d**) and quantification (**e**) of CD31^+^Sca-1^high^ EC-lined vessels (i.e., arterioles) in the tibias from wt, *miR-126*^*ECΔ/Δ*^ and *Spred1*^*ECΔ/Δ*^ mice. One of the three independent experiments with similar results is shown. Yellow arrows indicate CD31^+^Sca-1^high^ EC-lined vessels; white arrows indicate CD31^+^Sca-1^low^ EC-lined vessels. **f** and **g** Representative plots (**f**) and aggregate results (**g**) of Sca-1^high^ and Sca-1^low^ subfractions in BM ECs from wt, *miR-126*^*ECΔ/Δ*^ and *Spred1*^*ECΔ/Δ*^ mice by flow cytometry analysis (*n* = 3 mice per group). **h** CD31 (FITC) and Sca-1 (PE) IF staining (top) and quantification (bottom) of CD31^+^Sca-1^high^ EC-lined vessels (i.e., arterioles) in the tibias from normal wt mice treated with CpG-scramble (SCR) or CpG-miR-126 inhibitor (miRisten, 20 mg/kg/day, iv, for 3 weeks; *n* = 3 mice per group). Yellow arrows indicate CD31^+^Sca-1^high^ EC lined-vessels; white arrows indicate CD31^+^Sca-1^low^ EC-lined vessels. **i** Representative plots (left) and aggregate results (right) of Sca-1^high^ and Sca-1^low^ subfractions in BM ECs from normal wt mice treated with SCR or miRisten for 3 weeks (*n* = 3 mice per group). Results represent mean ± SEM. Significance values: *p < 0.05; **p < 0.01; ***p < 0.001; ****p < 0.0001

Interestingly, we noticed that miR-126 knock-down (KD) by miRZip anti-miR-126 lentiviral transduction (Fig. 3a, Additional file 1: Fig. S9a) or treatment with a CpG-miR-126 inhibitor [31] (an anti-miR-126 oligonucleotide hereafter called miRisten; Additional file 1: Fig. S9b, c) depleted the Sca-1^high^ fraction in CD31^+^Sca-1^high^ ECs from normal wt mice. Conversely, miR-126 over-expression (OE) in CD31^+^Sca-1^low^ ECs obtained by transduction with miR-126 precursor lentivirus (Fig. 3b) or treatment with CpG-miR-126 mimics (Additional file 1: Fig. S9d, e) enriched the Sca-1^high^ fraction. In vivo, mice with EC miR-126 KO (i.e., *miR-126*^*ECΔ/Δ*^; Fig. 3c, middle box) [12, 31] had fewer CD31^+^Sca-1^high^ ECs and arterioles than *miR-126*^*flox/flox*^*Tie2-cre-* controls (Fig. 3d–g, middle panel). Fluorescent reporter mice with tamoxifen-induced miR-126 KO in ECs [i.e., tamoxifen-treated *miR-126*^*flox/flox*^/Tie2-CreER/TdTomato/Tg(Ly6a-GFP)] also showed fewer Sca-1^high^ ECs (i.e., tdTomato^+^GFP^high^) and arterioles than wt reporter mice [i.e., tamoxifen-treated miR-126^wt/wt^/Tie2-CreER/TdTomato/Tg(Ly6a-GFP)] (Additional file 1: Fig. S9f, g). Furthermore, normal wt mice treated in vivo with miRisten (20 mg/kg/day, iv) for 3 weeks showed fewer CD31^+^Sca-1^high^ ECs and vessels (i.e., arterioles) than control mice treated with CpG-scramble (SCR) (Fig. 3h, i). In addition, similar to the leukemic mice, *miR-126*^*ECΔ/Δ*^ mice had an increase of the BM vessel permeability compared to wt controls (Additional file 1: Fig. S9h).

We previously reported that Spred1, a member of the Sprouty family of proteins and an inhibitor of RAS small GTPases, is both a miR-126 target and a regulator of miR-126 biogenesis [31]. EC Spred1 KO mice (i.e., *Spred1*^*f/f*^*Tie2-cre*+, hereafter called *Spred1*^*ECΔ/Δ*^) [13, 32, 33] therefore express constitutively higher levels of EC miR-126 than *Spred1*^*f/f*^*Tie2-cre*- and *miR-126*^*ECΔ/Δ*^ mice (Fig. 3c, right box) and represent a functional model for EC miR-126 OE. Accordingly, *Spred1*^*ECΔ/Δ*^ mice had more BM CD31^+^Sca-1^high^ ECs and vessels (i.e., arterioles) than wt controls (Fig. 3d–g, right panel).

Taken altogether, these results support a role of miR-126 in determining enrichment of the Sca-1^high^ EC fraction and in turn arteriolar density in the BM niche and a role of TNFα-dependent miR-126 downregulation in the loss of arterioles during leukemia growth.

**Fig. 4:**
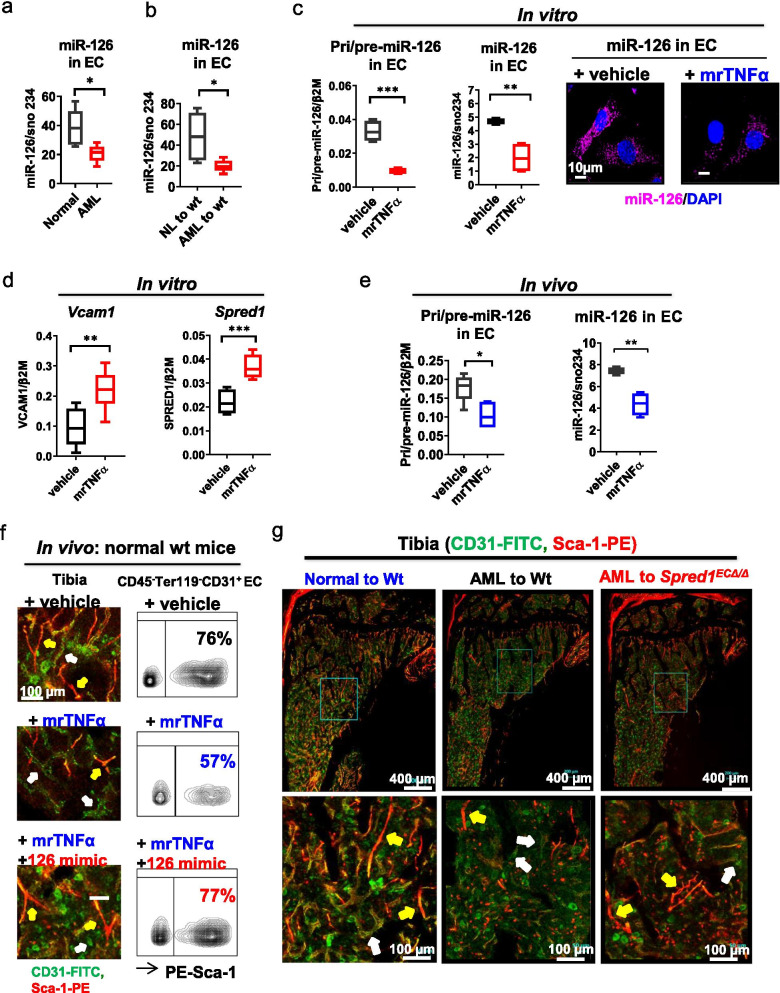
TNFα induced vascular remodeling of the leukemic BM niche via EC miR-126 downregulation. **a** miR-126 levels in BM ECs from normal wt and *Mll*^PTD/WT^/*Flt3*^ITD/ITD^ AML mice by Q-RT-PCR (*n* = 5 mice per group). **b** miR-126 levels in BM ECs from wt recipient mice engrafted with BM cells from normal wt or *Mll*^PTD/WT^/*Flt3*^ITD/ITD^ AML mice by Q-RT-PCR (*n* = 5 mice per group). **c** Levels of pri/pre- and mature miR-126 by Q-RT-PCR (*n* = 5 mice per group; left) and mature miR-126 expression by miRNA staining (right; one of three independent experiments with similar results is shown) in BM ECs from normal wt mice exposed in vitro to mrTNFα (1 ng/ml) or vehicle for 24 h. Scale bars represent a size of 10 µm. **d** Levels of *Vcam1* and *Spred1* in BM ECs from normal wt mice exposed in vitro to mrTNFα (1 ng/ml) or vehicle for 24 h by Q-RT-PCR (*n* = 5 mice per group). **e** Levels of pri/pre- (left) and mature miR-126 (right), as analyzed by Q-RT-PCR, in BM ECs from normal wt mice treated in vivo with mrTNFα (1 µg/day, ip, 3 weeks). **f** CD31 (FITC) and Sca-1 (PE) IF staining of tibias (left) and flow cytometry analysis of BM EC Sca-1^high^ and Sca-1^low^ subfractions (right) from normal wt mice treated in vivo with vehicle, mrTNFα (1 µg/day, ip), or mrTNFα+ miR-126 mimics (20 mg/kg/day, iv, 3 weeks; *n* = 3 mice per group). Yellow arrows indicate CD31^+^Sca-1^high^ EC lined-vessels; white arrows indicate CD31^+^Sca-1^low^ EC-lined vessels. Scale bars represent a size of 100 µm. One of three independent experiments with similar results is shown. **g** CD31 (FITC) and Sca-1 (PE) IF staining of tibias from normal wt recipient mice engrafted with BM MNCs from congenic normal mice (normal to wt) or leukemic mice (AML to wt) and of tibias from normal *Spred1*^*ECΔ/Δ*^ recipient mice engrafted with BM MNCs from congenic leukemic mice (AML to *Spred1*^*ECΔ/Δ*^). Yellow arrows indicate CD31^+^Sca-1^high^ EC lined-vessels; white arrows indicate CD31^+^Sca-1^low^ EC-lined vessels. One of three independent experiments with similar results is shown. Results represent mean ± SEM. Significance values: *p < 0.05; **p < 0.01; ****p < 0.0001

To this end, we also observed significantly lower BM EC miR-126 levels in the leukemic *Mll*^PTD/WT^/*Flt3*^ITD/ITD^ mice (Fig. 4a) in addition to fewer BM CD31^+^Sca-1^high^ ECs and vessels (i.e., arterioles; Fig. 1a, b; Additional file 1: Fig. S2a) compared with normal wt controls. Similar changes were observed in normal wt mice engrafted with AML BM MNCs compared with those engrafted with normal BM MNCs (Figs. 1c, d and 4b; Additional file 1: Fig. S2e). Furthermore, murine BM ECs obtained from normal wt mice treated in vitro with mrTNFα (1 ng/ml, 24 h) showed reduced levels of pri/pre- and mature miR-126 (Fig. 4c) and increased levels of miR-126 targets (i.e., *Vcam1* and *Spred1*) (Fig. 4d). Co-treatment with miR-126 mimics rescued the loss of the CD31^+^Sca-1^high^ fraction induced by mrTNFα (Additional file 1: Fig. S10a, b, right panel). TNFαR1 and TNFαR2 blocking Abs also rescued the mrTNFα-induced miR-126 downregulation (Additional file 1: Fig. S10c) and the reduction of the CD31^+^Sca-1^high^ EC fraction (Additional file 1: Fig. S7c).

Consistent with the in vitro results, normal wt mice that were given mrTNFα (1 µg/day, ip injection, 3 weeks) [25, 26] had a significant in vivo reduction of BM EC pri/pre- and mature miR-126 levels (Fig. 4e). As expected, these mice also showed a decrease in CD31^+^Sca-1^high^ ECs and vessels (i.e., arterioles) compared with vehicle-treated controls (Fig. 4f, middle panel; Additional file 1: Fig. S10d, e, middle panel). In vivo co-treatment with miR-126 mimics (20 mg/kg/day, iv. 3 weeks) rescued these changes (Fig. 4f, bottom panel; Additional file 1: Fig. S10d, e, right panel). Accordingly, normal wt mice engrafted with BM MNCs from *Mll*^PTD/WT^/*Flt3*^ITD/ITD^ AML donors had higher levels of BM TNFα (Additional file 1: Fig. S7d) and reduced levels of BM EC miR-126 (Additional file 1: Fig. S10f), in addition to a decrease in CD31^+^Sca-1^high^ ECs and arterioles (Fig. 1c, d; Additional file 1: Fig. S2e), compared with mice engrafted with BM MNCs from normal wt donors. These changes were rescued in vivo by blocking TNFα with anti-TNFα Ab (1 mg/day, ip, 4 times/week for 3 weeks) (Additional file 1: Fig. S10g–i). Similar results were observed in NSGS mice engrafted with human FLT3-ITD+ AML blasts compared with those engrafted with normal CB CD34^+^ cells (Fig. 1g, h; Additional file 1: Fig. S3, S7e and S10j).

Of note, *Spred1*^*ECΔ/Δ*^ mice (EC miR-126 OE, Fig. 3c) treated in vivo with mrTNFα (1 µg/day, ip, 3 weeks) showed no significant difference in vascularization compared with vehicle-treated *Spred1*^*ECΔ/Δ*^ controls (Additional file 1: Fig. S10k–l). Transplantation of AML BM MNCs resulted in a lesser degree of leukemia-induced BM vascular changes in *Spred1*^*ECΔ/Δ*^ recipients compared with wt recipients (Fig. 4g, right panel; Additional file 1: Fig. S10m, right panel), suggesting that constitutively higher expression of miR-126, as occurred in ECs from *Spred1*^*ECΔ/Δ*^ mice, may rescue in vivo TNFα-induced miR-126 depletion and loss of CD31^+^Sca-1^high^ ECs.

While the molecular mechanisms through which TNFα induces endothelial miR-126 downregulation are likely to be multifaceted, we focused on GATA2 since this protein is a verified miR-126 transcription factor [34–36] and is reportedly downregulated by TNFα [34, 37]. We first noticed that Gata2 levels were reduced in BM ECs from AML mice compared with normal mice (Additional file 1: Fig. S11a) and that GATA2 KD by siRNA in human umbilical vein ECs (HUVECs) decreased miR-126 levels (Additional file 1: Fig. S11b, c). We then demonstrated that in vitro*,* TNFα treatment (1 ng/ml) reduced Gata2 levels in both murine BM ECs and HUVECs (Additional file 1: Fig. S11d–f) as compared with vehicle-treated controls. Using chromatin immunoprecipitation assay, we showed a reduced GATA2 enrichment on the EGFL7/miR-126 promoter [34] in HUVECs exposed to TNFα (Additional file 1: Fig. S11g, h).

Taken altogether, these results support the notion that loss of CD31^+^Sca-1^high^ ECs and associated vessels (i.e., arterioles) observed during AML growth is at least partly mediated by EC miR-126 downregulation via TNFα-dependent decrease of GATA2 transcriptional activity.

### Antileukemic treatment restores CD31^+^Sca-1^high^ vessels that safeguard LSCs

Having demonstrated that TNFα secreted by the AML blasts contributes to loss of CD31^+^Sca-1^high^ ECs, we then reasoned that cytoreductive therapy that eliminates TNFα-secreting blasts could reverse the depletion of CD31^+^Sca-1^high^ EC-lined vessels (i.e., arterioles) as observed in the leukemic BM niche. To this end, we transplanted BM MNCs from *Mll*^PTD/wt^/*Flt3*^ITD/ITD^ AML mice (CD45.2) into congenic B6 mice (CD45.1) and generated a cohort of AML mice that developed disease at a similar time. We elected to treat these mice with TKIs rather than chemotherapy to restrict the observed BM niche changes to a direct blast cytoreduction rather than to non-specific chemotherapy cytotoxicity to other non-hematopoietic cells.

**Fig. 5:**
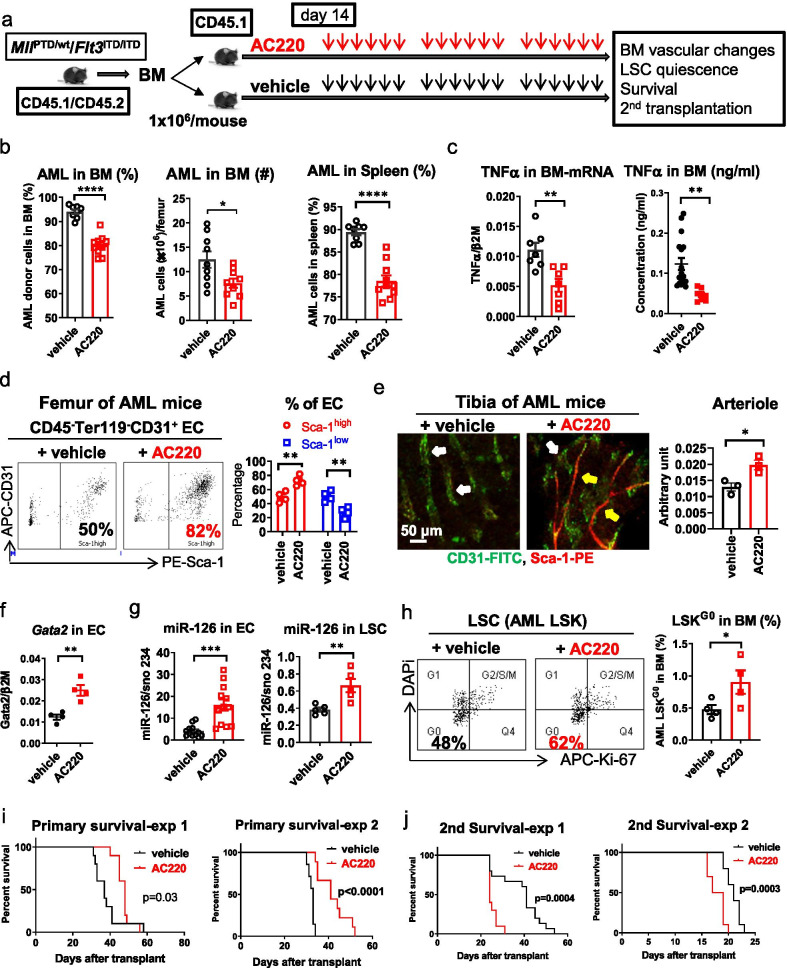
Treatment-induced CD31^+^Sca-1^high^ re-vascularization mediates LSC resistance. **a** Schematic design of the experiments. BM MNCs from diseased *Mll*^PTD/wt^/*Flt3*^ITD/ITD^ mouse (CD45.1/CD45.2) were transplanted into normal wt recipient mice (CD45.1, 6 Gy) to generate a cohort of leukemic mice with a similar disease onset time. At day 14 post transplantation, the mice were treated with AC220 (20 mg/kg/day, oral gavage) or vehicle for 3 weeks. After completion of treatment, a cohort of treated mice (*n* = 10 mice per group) were monitored for survival and another cohort of mice (*n* = 9 mice per group) were euthanized and assessed for BM vascular changes and LSC burden by 2nd transplantation. **b** Frequency and number of AML cells in BM and spleen from AC220-treated versus vehicle-treated AML mice. **c** TNFα mRNA expression in BM MNCs by Q-RT-PCR (left) and TNFα protein concentrations in BM plasma by Luminex assay (right) in AC220-treated versus vehicle-treated AML mice. **d** and **e** Long bones (femurs and tibias) from AML mice treated with vehicle or AC220 for 3 weeks were evaluated for: BM EC Sca-1^high^ and Sca-1^low^ subfractions (**d** left, representative plots; right, aggregate results) by flow cytometry analysis (*n* = 4 mice per group), and CD31^+^Sca-1^high^ EC-lined vessels (i.e., arterioles) by CD31 (FITC) and Sca-1 (PE) IF staining (**e** left) and quantification (**e** right) (*n* = 3 mice per group). For **e**: Yellow arrows indicate CD31^+^Sca-1^high^ EC-lined vessels; white arrows indicate CD31^+^Sca-1^low^ EC-lined vessels; scale bars represent a size of 50 µm. **f** Gata2 levels in BM ECs from AML mice treated with AC220 or vehicle for 3 weeks, analyzed by Q-RT-PCR (*n* = 4 mice per group). **g** miR-126 levels in BM ECs (left) and LSKs (right) from AML mice treated with vehicle or AC220 for 3 weeks, analyzed by Q-RT-PCR. **h** Representative plots of cell cycling of BM LSKs (left) and frequency of quiescent BM LSKs (i.e., LSKs in G0 phase, LSC^G0^, right) from AML mice treated with vehicle or AC220 for 3 weeks, analyzed by Ki-67 and DAPi staining and flow cytometry analysis. **i** and **j** Survival of AML mice treated with vehicle or AC220 for 3 weeks (primary, **i**) and survival of 2nd recipient mice (2nd survival, **j**) receiving BM cells from AC220-treated or vehicle-treated AML donors. Two of three independent experiments with similar results were shown. Results represent mean ± SEM. Significance values: *p < 0.05; **p < 0.01; ***p < 0.001; ****p < 0.0001

Upon confirmation of AML development at 4 weeks after transplantation, we treated these mice with the TKI AC220 [38] to target FLT3-ITD and cytoreduce AML blasts (20 mg/kg/day, oral gavage, Fig. 5a). After 3 weeks treatment, we observed leukemic cytoreduction (Fig. 5b) and decreased BM TNFα levels (Fig. 5c) in AC220-treated AML mice compared with vehicle-treated controls. In AC220-treated mice, we noticed a gain in BM CD31^+^Sca-1^high^ ECs and vessels (i.e., arterioles) (Fig. 5d, e), an increase of Gata2 levels in ECs (Fig. 5f) but not in LSKs (Additional file 1: Fig. S12a), an increase of miR-126 levels in both ECs and LSCs (i.e., AML LSK; Fig. 5g, Additional file 1: Fig. S12b), and a higher frequency of quiescent LSK cells (LSK^G0^, Fig. 5h; Additional file 1: Fig. S12c) compared with vehicle-treated controls.

In *Mll*^PTD/WT^/*Flt3*^ITD/ITD^ AML mice, BM LSKs are the subpopulation mostly enriched in leukemia-initiating cells (LICs; hereafter called LSCs) as shown by limiting-dilution transplantation of immunophenotypically defined BM cell subpopulations (Additional file 1: Fig. S12d). To this end, we showed that while AC220 effectively cytoreduced AML blasts and increased survival of the primary treated mice (Fig. 5i), it also unexpectedly increased LSC burden and/or activity. In fact, in secondary (2nd) transplant experiments, recipients of BM MNCs from AC220-treated donors had a higher disease burden (Additional file 1: Fig. S12e) and shorter survival (Fig. 5j) than recipients of BM MNCs from vehicle-treated donors, indicating not only persistence but also an expansion of LSCs.

**Fig. 6:**
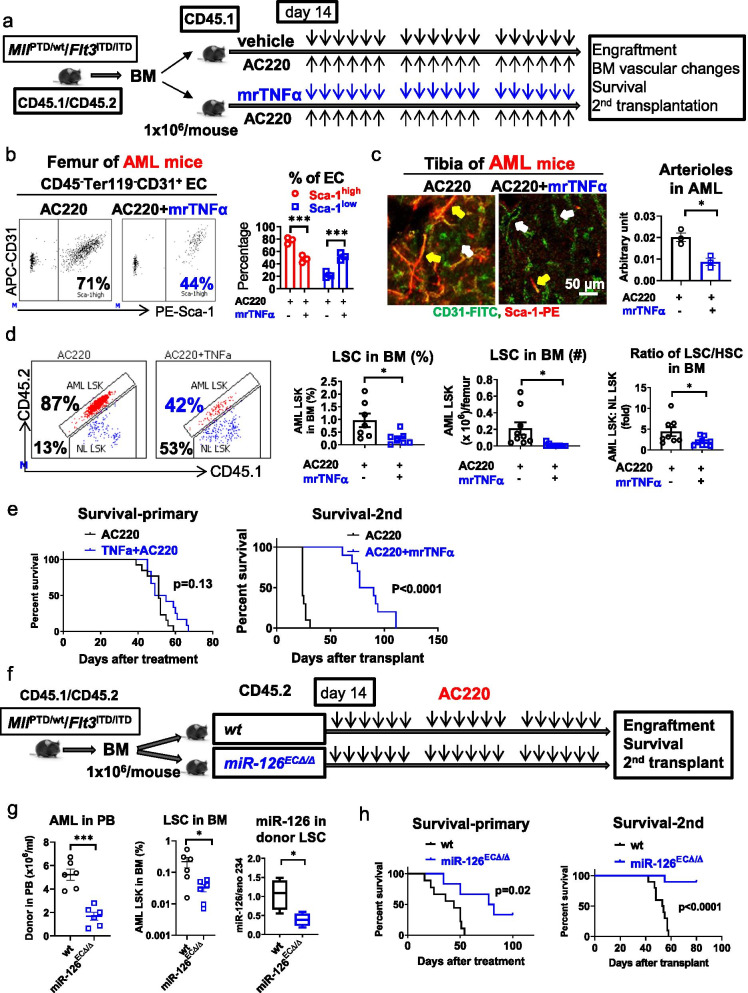
Preventing treatment-induced CD31^+^Sca-1^high^ re-vascularization enhances LSC sensitivity to TKI. **a** Schematic design of the experiments. BM MNCs from diseased *Mll*^PTD/wt^/*Flt3*^ITD/ITD^ AML mice (CD45.1/CD45.2) were transplanted into normal wt recipient mice (CD45.1, 6 Gy) to generate a cohort of mice with a similar disease onset time. At day 14 post transplantation, the leukemic mice were treated with AC220 (20 mg/kg/day, oral gavage)+ mrTNFα (1 µg/day, ip) or AC220+ vehicle for 3 weeks. After completion of treatment, a cohort of treated mice (*n* = 12 mice per group) were monitored for survival and another cohort of mice (*n* = 7 mice per group) were euthanized and assessed for BM vascular changes and LSC burden by 2nd transplantation. **b** and **c** Long bones (femurs and tibias) from the above leukemic mice treated with AC220+ mrTNFα or AC220+ vehicle for 3 weeks (*n* = 3 mice per group) were evaluated for: BM EC Sca-1^high^ and Sca-1^low^ subfractions (**b** left, representative plots; right, aggregate results) by flow cytometry analysis, and CD31^+^Sca-1^high^ EC-lined vessels (i.e., arterioles) by CD31 (FITC) and Sca-1 (PE) IF staining (**c** left) and quantification (**c**, right). For **c**: Yellow arrows indicate CD31^+^Sca-1^high^ EC-lined vessels; white arrows indicate CD31^+^Sca-1^low^ EC-lined vessels; scale bars represent a size of 50 µm. **d** Representative plots (left) and aggregate results of frequency and number of LSCs (i.e., CD45.1/CD45.2 LSKs, middle), and ratio of LSCs/HSCs (i.e., CD45.1/CD45.2 AML LSKs: CD45.1 normal LSKs, right) in the BM from AC220+ mrTNFα-treated and AC220+ vehicle-treated leukemic mice. **e** Survival of treated primary AML mice (left) and 2nd recipient mice (right) receiving BM cells from AC220+ mrTNFα-treated or AC220+ vehicle-treated AML donors. **f** Schematic design of the experiments. BM MNCs from diseased *Mll*^PTD/wt^/*Flt3*^ITD/ITD^ mice (CD45.1/CD45.2) were transplanted into wt and EC miR-126 KO (*miR-126*^*ECΔ/Δ*^*)* recipient mice (CD45.2, 6 Gy) and evaluated for response to AC220 (20 mg/kg/day, oral gavage) given for 3 weeks. **g** Circulating leukemia burden (left) and frequency of BM LSCs (CD45.1/CD45.2 LSKs, middle) by flow cytometry analysis and miR-126 levels in LSCs by Q-RT-PCR (right) from leukemic miR-126 wt and *miR-126*^*ECΔ/Δ*^ mice treated with AC220 for 3 weeks. **h** Survival of AC220-treated leukemic miR-126 wt and *miR-126*^*ECΔ/Δ*^ primary mice (left) and 2nd recipients (right, *n* = 10 mice per group) of BM cells from the AC220-treated leukemic miR-126 wt and *miR-126*^*ECΔ/Δ*^ mice. Results represent mean ± SEM. Significance values: *p < 0.05; **p < 0.01; ***p < 0.001; ****p < 0.0001

In contrast, AC220-treated mice that also received mrTNFα (1 µg/day, ip, 3 weeks) to prevent the treatment-related TNFα decrease (Fig. 6a) maintained lower levels of BM EC miR-126 (Additional file 1: Fig. S12f) and had fewer CD31^+^Sca-1^high^ ECs and arterioles (Fig. 6b, c) and less LSCs (i.e., CD45.1/CD45.2 AML LSKs; Fig. 6d; Additional file 1: Fig. S12b) than mice that received AC220 alone. In 2nd transplant experiments, the recipients of BM MNCs from AC220+ mrTNFα-treated donors had a significantly longer survival compared with the recipients of BM MNCs from AC220-treated donors (Fig. 6e, right; median survival: 84 vs. 24 days, *p* < 0.0001), supporting the hypothesis that prevention of treatment-related CD31^+^Sca-1^high^ EC enrichment and arteriolar re-vascularization prevents LSC expansion. We confirmed the relevance of these results to human disease using the FLT3-ITD+ PDX model (Additional file 1: Fig. S12g–k).

Next, as TNFα acts on the vasculature of the BM niche via miR-126, we reasoned that a direct EC miR-126 deprivation of the BM niche could prevent treatment-induced CD31^+^Sca-1^high^ EC enrichment, arteriolar re-vascularization and LSC protection. To this end, we transplanted BM MNCs from *Mll*^PTD/wt^/*Flt3*^ITD/ITD^ AML mice into *miR-126*^*ECwt/wt*^ and *miR-126*^*ECΔ/Δ*^ recipient mice and treated them with AC220 (20 mg/kg/day, oral gavage; Fig. 6f). After 3 weeks treatment, we observed reduced CD31^+^Sca-1^high^ ECs and arterioles (Additional file 1: Fig. S13a–d), a significant reduction of leukemia burden and LSCs, reduced miR-126 levels in AML LSCs (Fig. 6g) likely due to reduced EC supply, and increased survival (Fig. 6h; left) in the AC220-treated *miR-126*^*ECΔ/Δ*^ primary mice compared with the AC220-treated *miR-126*^*ECwt/wt*^ controls. BM MNCs from AC220-treated *miR-126*^*ECΔ/Δ*^ or *miR-126*^*ECwt/wt*^ AML donors were then transplanted into 2nd wt recipient mice. Recipients of BM MNCs from the AC220-treated *miR-126*^*ECΔ/Δ*^ donors had a significantly longer survival than recipients of BM MNCs from the AC220-treated *miR-126*^*ECwt/wt*^ donors (Fig. 6h, right; median survival: 53 days vs. not reached at day 80 post transplantation, *p* < 0.0001).

Similar results were obtained by pharmacologic miR-126 downregulation with miRisten. A cohort of *Mll*^PTD/WT^/*Flt3*^ITD/ITD^ leukemic mice were generated as described above and then treated with miRisten (20 mg/kg, iv, daily), SCR, miRisten+ AC220 (10 mg/kg, oral gavage, daily), or SCR+ AC220 for 3 weeks, followed by assessment of donor AML cell engraftment in PB, BM and spleen (Additional file 1: Fig. S14a). We confirmed miR-126 KD in ECs from miRisten-treated mice compared with SCR-treated mice (Additional file 1: Fig. S14b). Mice receiving the combination of miRisten+ AC220 had a significant reduction in the percentage of AML cells in PB, BM and spleen (Additional file 1: Fig. S14c, d), a significant decrease in the frequency of BM AML LSKs (Additional file 1: Fig. S14e) and increased survival (Additional file 1: Fig. S14f) compared to mice treated with SCR+ AC220. Similar to the mrTNFα+ AC220 combination, the miRisten+ AC220 combination also significantly prolonged survival in 2nd transplant experiments (Additional file 1: Fig. S14g; median survival: 37 days vs. not reached at day 65 post transplantation, *p* < 0.0001), suggesting reduced post-treatment LSC burden.

These results were corroborated in NSGS mice transplanted with FLT3-ITD+ AML blasts and then treated with miRisten+ AC220 or SCR+ AC220 for 3 weeks (Additional file 1: Fig. S15a). Treatment with miRisten+ AC220 significantly reduced human CD45^+^CD33^+^ cell engraftment (Additional file 1: Fig. S15b) and prolonged survival (Additional file 1: Fig. S15c, left) in the primary treated mice. Recipient mice receiving BM cells from miRisten+ AC220-treated donors lived significantly longer than the mice receiving BM cells from SCR+ AC220-treated donors (Additional file 1: Fig. S15c, right; median survival: 167 vs. 126 days, *p* < 0.0001), suggesting that the combination of miRisten+ AC220 had decreased the LSC burden.

## Discussion

AML cells reportedly produce inflammatory cytokines that profoundly remodel the BM vascular niche and create a microenvironment supportive of competitive leukemia growth over normal hematopoiesis [31, 39, 40]. However, how the leukemic BM niche adapts to the changing conditions caused by treatment and ultimately impacts on clinical outcome remains to be fully elucidated. Herein, we utilized FLT3-ITD+ AML murine and PDX models to study the vascular changes of the leukemic BM niche that occur upon molecular targeting of the AML blasts. During AML growth, in the leukemic BM niche, we observed a loss in CD31^+^Sca-1^high^ ECs, which line mainly non-permeable arterioles, and a gain in CD31^+^Sca-1^low^ ECs, which line mainly fenestrated, permeable sinusoids. These vascular changes were caused partly by high levels of TNFα produced by the AML blasts, causing downregulation of miR-126 in CD31^+^Sca-1^high^ ECs, which became depleted while an enrichment in CD31^+^Sca-1^low^ ECs was observed.

TNFα has been intensively studied for its role in normal and malignant hematopoiesis [41]. Yamashita and Passegue recently reported on the complex role of TNFα in normal and clonal hematopoiesis, showing that while inducing myeloid progenitor apoptosis, TNFα promotes HSC survival [42]. AML blasts express high levels of TNFα [43], and likely hijack TNFα-driven mechanisms of normal hematopoiesis to support leukemia growth [42]. Herein, we show a novel pro-leukemogenic role of TNFα that is extrinsic to AML cells and involves downregulation of miR-126 in the vascular compartment of the BM niche. While TNFα has been implicated in the remodeling of blood vessels and shown to promote angiogenesis during inflammation [44, 45], to our knowledge the TNFα-induced switch from Sca-1^high^ ECs to Sca-1^low^ ECs, and in turn from an arteriole- to a sinusoid-enriched BM niche as observed during leukemia growth has not been previously reported. As these changes were a phenocopy of genetic (EC miR-126 KO) and pharmacologic (i.e., miRisten) EC miR-126 deprivation, we postulated and proved that loss of CD31^+^Sca-1^high^ ECs and arterioles were due to TNFα-induced miR-126 downregulation.

MiR-126 is one of the most highly expressed microRNAs in ECs, where it acts as a master regulator of angiogenesis [12, 28, 29]. In developmental vasculogenesis, miR-126 supports differentiation of embryonic stem cells into endothelial precursor cells and mature ECs [46]. In mature ECs, miR-126 contributes to the maintenance of quiescence and vascular integrity and inhibition of endothelial permeability and apoptosis [12, 28, 29]. Furthermore, miR-126 enhances the activity of angiopoietin-1 (Ang-1), a glycoprotein that regulates vessel stabilization, maturation and permeability [47, 48]. Of note, the molecular mechanisms through which TNFα induces endothelial miR-126 downregulation are likely to be multifaceted and remain to be fully elucidated. Herein, we showed that they likely involve GATA2 [34, 35], a miR-126 transcription factor, which is reportedly silenced by TNFα [37]. But how do the TNFα-induced vascular changes in the leukemic BM niche ultimately promote leukemia growth? We previously showed that CD31^+^Sca-1^high^ ECs expressed the highest levels of miR-126 in the BM niche and supply miR-126 to maintain LSC quiescence [31]. Thus, by inducing loss of CD31^+^Sca-1^high^ ECs, TNFα decreases the endothelial supply of miR-126 to LSCs and enables them to enter the cell cycle and partially differentiate into proliferating “bulk” AML blasts [31, 49].

Of note, these observations may have direct translational and clinical relevance. In fact, under these conditions, therapeutic cytoreduction of AML blasts can cause a drop in the BM levels of TNFα and in turn lead to a post-treatment enrichment of BM CD31^+^Sca-1^high^ ECs and arterioles with a consequent increase in the endothelial miR-126 supply to LSCs, which, once enriched in miR-126, are more resistant to therapy[31, 49]. We proved this model in FLT3-ITD+ AML murine and PDX mice treated with TKIs. We showed that after TKI treatment, an increase in CD31^+^Sca-1^high^ ECs and arterioles occurred and LSCs persisted as demonstrated using secondary transplant experiments. Of note, LSC persistence could be prevented by blocking the gain in CD31^+^Sca-1^high^ ECs and the arteriolar “re-vascularization” of the BM niche with administration of recombinant TNFα or with deprivation of endothelial miR-126 via genetic EC miR-126 KO or pharmacological treatment with the anti-miR-126 miRisten.

In comparing our results with recent reports on the BM vascular changes occurring during AML growth and treatment, we found interesting similarities between these studies and our work [17, 39]. While Passaro et al. showed that engraftment of AML blasts in mouse BM increased CD31^+^Sca-1^+^ ECs [17], in line with our results, Duarte et al. reported that AML growth caused loss of CD31^+^Sca-1^+^ ECs in the BM niche [39]. Similar to our observation, both studies demonstrated an increase in BM vascular permeability during leukemia growth, which associates with CD31^+^Sca-1^low^ vessels. While these studies showed that the post-treatment changes rescued AML-induced BM permeability and enhanced sensitivity of the proliferating blasts to chemotherapy, they did not test the effects of these changes on LSC activity as done in our study. To our knowledge, we are the first to show that, in the BM niche, therapeutic cytoreduction of proliferating blasts decreases TNFα levels and leads to enrichment in CD31^+^Sca-1^high^ arterioles that safeguard LSCs. Of note, using PDX model transplanted with human FLT3-ITD+ AML cells, Maifrede et al. showed that AC220 (10 mg/kg/day, 7 days) prolonged survival of primary treated mice, but did not change survival of 2nd recipient mice [50]. Herein, using FLT3-ITD+ AML murine model, we showed that, treatment with AC220 (20 mg/kg/day, 21 days) prolonged survival of primary treated mice and resulted in a more severe disease in 2nd recipient mice (i.e., shorter survival). These results are not in conflict as in both studies, TKI treatment failed to reduce LSC burden. The difference in survival of secondary transplanted mice observed in these two studies could be related to technical differences including the used models and the schedule (7 vs. 21 days) and dosage (10 vs. 20 mg/kg/day) of the drug administration.

Thus, taken altogether, these results provide the evidence for non-genetic, LSC extrinsic mechanisms of treatment resistance that depend on the vascular plasticity of the leukemic BM niche and likely exemplify a treatment-related Janus phenomenon in AML [51]. Janus was an ancient Roman god with two faces looking in opposite directions. The two opposing “faces” of the TKI treatments in FLT3-ITD+ AML models as described here are the initially beneficial cytoreduction of proliferating blasts followed by post-treatment CD31^+^Sca-1^high^ EC gain and arteriolar re-vascularization of the BM niche that safeguard LSCs thereby creating the condition for disease relapse. Of note, it is likely that the “Janus” phenomenon that we report here for TKIs may also apply to other antileukemic therapies that target proliferating blasts but do not kill LSCs. To this end, prevention of post-treatment CD31^+^Sca-1^high^ EC expansion, arteriolar re-vascularization and LSC protection is achievable with pharmacological deprivation of BM endothelial miR-126 (i.e., miRisten), which may represent a novel strategy to overcome non-genetically driven, extrinsic mechanisms of LSC resistance in AML.

## Conclusions

The TNFα-miR-126 axis plays a key role in the BM vascular changes induced by antileukemic treatments and mediates non-genetic, extrinsic mechanisms of LSC treatment-resistance that can be overcome with pre-emptive therapeutic deprivation of EC miR-126.

## Supplementary Information

**Additional file 1.** Treatment-induced Arteriolar Revascularization and miR-126 Enhancement in Bone Marrow Niche Protect Leukemic Stem Cells in AML.

## Data Availability

RNA sequencing data produced in our laboratory and analysed in this study are available at the Gene Expression Omnibus (GEO) repository of the National Center for Biotechnology Information (GSE180104). Supplementary information including Additional file 1: Figs. S1–S15 and Tables S1–S3 are provided with the online version of this paper. All other datasets generated during this study are available from the corresponding author on reasonable request.

## Abbreviations

AML: Acute myeloid leukemia
BM: Bone marrow
HSC: Hematopoietic stem cell
LSC: Leukemic stem cell
EC: Endothelial cell
TNFα: Tumor necrosis factor alpha
PB: Peripheral blood
MNC: Mononuclear cell
COHNMC: City of Hope National Medical Center
FLT3: FMS-like tyrosine kinase 3
ITD: Internal tandem duplication
TK: Tyrosine kinase
TKI: Tyrosine kinase inhibitor
wt: Wild type
CB: Cord blood
PDX: Patient-derived xenograft
L^−^S^−^K^−^: Lin^−^Sca-1^−^c-Kit^−^
LSK: Lin^−^Sca-1^+^c-Kit^+^
LT-HSC: Long-term hematopoietic stem cell
mr: Murine recombinant
Ab: Antibody
OB: Osteoblast
MSP: Mesenchymal stromal progenitor
KO: Knockout
KD: Knock-down
HUVEC: Human umbilical vein endothelial cells
2nd: Secondary
Ang-1: Angiopoietin-1
