# Supplementary material for: Treatment-induced arteriolar revascularization and miR-126 enhancement in bone marrow niche protect leukemic stem cells in AML
Source: J Hematol Oncol. 2021 Aug 9;14:122. doi: 10.1186/s13045-021-01133-y (PMC8351342; doi:10.1186/s13045-021-01133-y)
Supplement: Supplementary file 1 — Additional file 1. Treatment-induced Arteriolar Revascularization and miR-126 Enhancement in Bone Marrow Niche Protect Leukemic Stem Cells in AML. [file 13045_2021_1133_MOESM1_ESM.docx]

**Supplementary Information**

**Additional file 1**

**Figure S1**. BM vascular staining of normal mice

**Figure S2**. Vascular remodeling of the leukemic BM niche in AML murine model

**Figure S3**. Vascular remodeling of the leukemic BM niche in AML PDX model

**Figure S4**. Vessel permeability in calvarial BM of normal Sca-1 reporter mouse

**Figure S5**. Increased vessel permeability in AML BM compared to normal BM

**Figure S6**. Levels of cytokines in the BM and blood of normal wt and FLT3-ITD+ AML mice

**Figure S7**. TNFα mediates decrease of CD31^+^Sca-1^high^ EC-lined vessels in the BM niche of FLT3-ITD+ AML

**Figure S8**. Gene expression profiling of Sca-1^high^ and Sca-1^low^ ECs from normal wt mice

**Figure S9**. miR-126 maintains CD31^+^Sca-1^high^ ECs in the BM niche

**Figure S10**. TNFα-miR-126 axis mediates vascular remodeling of the leukemic BM niche

**Figure S11**. Gata2 mediates TNFα-induced miR-126 downregulation in ECs

**Figure S12**. Preventing treatment-induced BM vascular changes by maintaining BM TNFα levels rescues LSC treatment-resistance

**Figure S13**. BM vascularization in AC220-treated miR-126^ECΔ/Δ^ versus miR-126 wt leukemic mice

**Figure S14**. Preventing treatment-induced BM vascular changes by miRisten rescues LSC treatment-resistance in AML murine model.

**Figure S15**. Preventing treatment-induced BM vascular changes by miRisten rescues LSC treatment-resistance in AML PDX model.

**Table S1**. Information of AML patient samples used in this study

**Table S2**. Gene ontology analysis of differentially expressed genes in Sca-1^high^ ECs vs Sca-1^low^ ECs

**Table S3**. Taqman gene assay list

**Supplementary materials and methods**

**Figure S1.** **BM vascular staining of normal mice. a-e,** CD31 (FITC) and α-SMA (PE) (**a**), α-SMA (FITC) and Sca-1 (PE) (**b**), α-SMA (PE) and Endomucin (Emcn, FITC) (**c**), CD31 (FITC) and Sca-1 (PE) (**d**), Sca-1 (PE) and Emcn (FITC) (**e**) IF staining and confocal imaging of tibia from normal wt mouse. Blue arrows indicate CD31+α-SMA+ (**a**), α-SMA+Sca-1^high^ (**b**) and α-SMA+Emcn- (**c**) EC-lined vessels (i.e., artery); yellow arrows indicate CD31+α-SMA- (**a**), α-SMA-Sca-1^high^ (**b**), CD31+Sca-1^high^ (**d**) and Sca-1^high^Emcn- (**e**) EC-lined vessels (i.e., arteriole); white arrows indicate CD31+α-SMA- (**a**), α-SMA-Emcn+ (**c**), CD31+Sca-1^low^ (**d**) and Sca-1^low^Emcn+ (**e**) EC-lined vessels (i.e., sinusoid). Scale bar represents a size of 100µm. **f,** Confocal imaging of EC (tdTomato+) and Sca-1 (GFP+) in tibia from tamoxifen-administrated Tie2-CreER/TdTomato/Tg(Ly6a-GFP) reporter mouse. Blue arrow indicates Sca-1^high^ EC-lined artery; yellow arrow indicates Sca-1^high^ EC-lined arteriole; white arrow indicates Sca-1^low^ EC-lined sinusoid; scale bar represents a size of 100µm. For **a-f**: One of the three independent experiments with similar results is shown.

**Figure S2. Vascular remodeling of the leukemic BM niche in AML murine model. a,** CD31 (FITC) and Sca-1 (PE) IF staining of tibias from normal wt and AML mouse (n=3 mice per group), relative to Fig. 1**a**. **b-c,** Representative plots (**b**) and aggregate results of percentage and number (**c**) of CD45^-^Ter119^-^CD31^+^ ECs in the BM of normal wt and AML mice, analyzed by flow cytometry. **d,** Representative plots of flow cytometry analysis showing gating strategy of Sca-1^high^ and Sca-1^low^ fraction in BM CD45^-^Ter119^-^CD31^+^ ECs, relative to Fig. 1**b**. **e,** CD31 (FITC) and Sca-1 (PE) IF staining of tibias from normal wt recipient mice engrafted with BM cells from normal wt (Normal to wt) or AML (AML to wt) mice, relative to Fig. 1**c**. **f,** Confocal imaging of EC (tdTomato+) and Sca-1 (GFP+) in tibias from tamoxifen-treated Tie2-CreER/TdTomato/Tg(Ly6a-GFP) reporter mice engrafted with BM MNCs from normal wt (Normal to reporter) or leukemic *Mll*^PTD/WT^/*Flt3*^ITD/ITD^ (AML to reporter) mice, relative to Fig. 1**e**. **g,** Representative plots of flow cytometry analysis showing gating strategy of Sca-1^high^ (TdTomato^+^GFP^high^) and Sca-1^low^ (TdTomato^+^GFP^low^) fraction in BM CD45^-^Ter119^-^CD31^+^ ECs from the reporter mice, relative to Fig. 1**f**. For **a** and **e**: yellow arrows indicate CD31^+^Sca-1^high^ EC-lined vessels (i.e., arteriole); white arrows indicate CD31^+^Sca-1^low^ EC-lined vessels (i.e., sinusoid); for **f**: yellow arrows indicate Sca-1^high^ EC-lined vessels (TdTomato^+^GFP^high^); white arrows indicate Sca-1^low^ EC-lined vessels (TdTomato^+^GFP^low^); scale bars represent a size of 400µm. Results represent mean±SEM. Significance values: *, p<0.05; **, p<0.01.

**Figure S3. Vascular remodeling of the leukemic BM niche in AML PDX model.** CD31 (FITC) and Sca-1 (PE) IF staining of tibias from NSGS mice engrafted with CB CD34^+^ cells or human FLT3-ITD+ AML blasts, relative to Fig. 1**g**. Yellow arrows indicate CD31^+^Sca-1^high^ EC-lined vessels (i.e., arteriole); white arrows indicate CD31^+^Sca-1^low^ EC-lined vessels (i.e., sinusoid); scale bars represent a size of 400µm.

**Figure S4. Vessel permeability in calvarial BM of normal Sca-1 reporter mouse.** Demonstration of vessel permeability as shown by dextran (labeled with TRITC,150kDa, red) leakiness out of vessels measured at 30 minutes (min) post dextran administration in the calvaria of normal Sca-1 (GFP) reporter mice using intravital confocal microscopy. Yellow arrows indicate no obvious leakiness (i.e., no diffuse dextran staining) outside GFP-Sca-1^high^ EC-lined vessels (i.e., arterioles); white arrows indicate more pronounced leakiness (i.e., diffuse dextran staining) outside GFP-Sca-1^low^ EC-lined vessels (i.e., sinusoids). One of three independent experiments with similar results is shown.

**Figure S5. Increased vessel permeability in AML BM compared to normal BM.** **a-b,** Vessel permeability as shown by dextran leakiness out of calvarial vessels in normal wt and AML mice (**a,** labeled with TRITC-150kDa, red) and in NSGS mice transplanted with normal cord blood (CB) CD34^+^ cells or human FLT3-ITD+ AML blasts (PDX) (**b,** labeled with FITC,150kDa, green), assessed at 30 minutes (min) post dextran administration using intravital confocal microscopy. White arrows indicate representative regions of leakiness (i.e., diffuse dextran staining) observed mainly outside the vessels from the AML mice or PDXs. **c-d,** Vessel permeability as shown by dextran (labeled with FITC,150kDa, green) leakiness out of calvarial vessels of wt (**c**) and tamoxifen-treated Tie2-CreER/TdTomato (EC reporter) (**d**) mice transplanted with normal (NL to wt or NL to EC reporter) or AML (AML to wt or AML to EC reporter) cells (engraftment rates >70%), assessed at 30 min (**c**), or 20, 40, 60 and 80 min (**d**), post-dextran administration using intravital confocal microscopy. White arrows indicate representative regions of leakiness observed mainly outside the vessels from the wt or reporter mice transplanted with AML cells (i.e., AML to wt or AML to EC reporter). **e,** Vessel permeability as shown by dextran (labeled with Alex647, 150kDa, blue) leakiness out of calvarial vessels of normal (top) and AML-bearing (bottom) double reporter mice (i.e., tamoxifen-treated Tie2-CreER/TdTomato/Tg(Ly6a-GFP), assessed at 30 min post dextran administration using intravital confocal microscopy. Yellow arrows indicate no obvious leakiness out of GFP-Sca-1^high^ EC-lined vessels; white arrows indicate representative regions of leakiness observed mainly outside the GFP-Sca-1^low^ EC-lined vessels from the AML-bearing reporter mice. **a-e**: one of three independent experiments with similar results is shown.

**Figure S6. Levels of cytokines in the BM and blood of normal wt and FLT3-ITD+ AML mice. a-m,** Levels of G-CSF (**a**), EOTAXIN (**b**), IL-1α (**c**), IL-1β (**d**), IL-6 (**e**), IL-9 (**f**), GM-CSF (**g**), IP-10 (**h**), Vegfα (**i**), KC (**j**), MIG (**k**), MIP-2 (**l**) and RANTES (**m**) in the BM and blood of wt and AML mice, as analyzed by Luminex assay. Levels of IFN-γ, IL-2, IL-3, IL-4, IL-5, IL-7, IL-10, IL-12 (p40), IL-12 (p70), IL-13, IL-15, IL-17, LIF, LIX, MCP-1, M-CSF, MIP-1α and MIP-1β were undetectable. Results represent mean±SEM. Significance values: *, p<0.05; **, p<0.01; ***, p<0.001; ****, p<0.0001; ns, not significant.

**Figure S7. TNFα mediates decrease of CD31^+^Sca-1^high^ EC-lined vessels in the BM niche of FLT3-ITD+ AML.** **a,** Frequency of Sca-1^high^ and Sca-1^low^ subfractions in BM ECs from normal wt mice exposed *in vitro* to mrTNFα (i.e., 0.2, 1, 10ng/ml) for 96 hrs, analyzed by flow cytometry (n=4). **b,** Flow cytometry analysis of TNFαR1 and TNFαR2 expression on Sca-1^high^ and Sca-1^low^ BM ECs from wt and AML mice. One of the three independent experiments with similar results is shown. **c,** Sca-1 expression by flow cytometry analysis of BM ECs from normal wt mice exposed *in vitro* to vehicle, mrTNFα (1ng/ml), mrTNFα+anti-TNFαR1 (1µg/ml), mrTNFα+anti-TNFαR2 (1µg/ml), or mrTNFα+anti-TNFαR1+anti-TNFαR2 for 96 hrs. One of the three independent experiments with similar results is shown. **d,** Levels of TNFα (pg/ml) in the BM plasma of wt recipient mice engrafted with BM MNCs from normal wt or AML donors, analyzed by Luminex assay (n= 9 mice per group). **e,** TNFα expression in the BM of NSGS mice at 12 wks post transplantation (engraftment rates > 70%) with normal CB CD34^+^ cells (1x10^5^/mouse) or human FLT3-ITD+ AML blasts (2x10^6^/mouse), as analyzed by immunohistochemistry (IHC). One of the three independent experiments with similar results is shown. Scale bars represent a size of 10µm. **f,** Frequency of BM ECs in NSGS mice engrafted with normal CB CD34^+^ cells or human FLT3-ITD+ AML blasts (n=4 mice per group, engraftment rates >70%). Results represent mean±SEM. Significance values: *, p<0.05; **, p<0.01; ***, p<0.001; ****, p<0.0001.

**Figure S8. Gene expression profiling of BM Sca-1^high^ and Sca-1^low^ ECs from normal wt mice. a,** Representative plots of flow cytometry analysis showing gating strategy of BM ECs (CD45^-^Ter119^-^CD31^+^), osteoblasts (OBs; CD45^-^Ter119^-^CD31^-^CD166^+^Sca-1^-^), mesenchymal stromal progenitors (MSPs; CD45^-^Ter119^-^CD31^-^CD166^-^Sca-1^+^), and long-term (LT-) HSCs [Lin^-^Sca-1^+^c-kit^+^ (LSK) Flt3^-^CD150^+^CD48^-^] from normal wt mice. **b,** Levels of miR-126 in BM ECs, LT-HSCs, OBs and MSPs from normal wt mice by Q-RT-PCR. **c,** Levels of pri/pre- (left) and mature miR-126 (right) in Sca-1^high^ and Sca-1^low^ ECs from the BM of normal wt mice by Q-RT-PCR. **d,** Mature miR-126 expression in Sca-1^high^ and Sca-1^low^ ECs from the BM of normal wt mice by miRNA staining. One of the three independent experiments with similar results is shown. **e,** Levels of the miR-126 target genes *Vcam1* and *Spred1* in Sca-1^high^ and Sca-1^low^ ECs from the BM of normal wt mice by Q-RT-PCR. **f,** Heatmap of differently expressed genes (DEGs, 4451 genes) in BM Sca-1^high^ (n=4) and Sca-1^low^ (n=5) EC subpopulations selected and pooled from normal wt mice (n=10), analyzed by RNA sequencing. **g,** Cluster analysis of differential gene expression. Heatmap for hierarchical clustering of a panel of selected genes (39 genes, Table S3) important in EC function in BM Sca-1^high^ versus Sca-1^low^ ECs selected from normal wt mice analyzed by RNA sequencing (left) or by Q-RT-PCR (right; relative to ß2m).

**Figure S9. miR-126 maintains CD31^+^Sca-1^high^ ECs in the BM niche. a,** Representative plots of flow cytometry analysis showing gating strategy of Sca-1^high^ and Sca-1^low^ fraction in cultured ECs, relative to Fig. 3**a**. **b,** miR-126 levels in cultured murine BM Sca-1^high^ ECs from normal wt mice assessed at 4 hrs post-treatment with SCR or miRisten (500nM) by Q-RT-PCR. **c,** Sca-1^low^ subfraction following exposure of Sca-1^high^ ECs from normal wt mice to SCR or miRisten (500nM) for 4, 16, 24 and 48 hrs, analyzed by flow cytometry. One of the three independent experiments with similar results is shown. **d,** miR-126 levels in cultured murine BM Sca-1^low^ ECs from normal wt mice assessed at 4 hrs post-treatment with SCR or miR-126 mimics (2µM) by Q-RT-PCR. **e,** Sca-1^high^ subfraction following exposure of Sca-1^low^ ECs from normal wt mice to SCR or miR-126 mimics (2µM) for 96 hrs, analyzed by flow cytometry. One of the three independent experiments with similar results is shown. **f,** Confocal imaging (left) and quantification (right) of Sca-1^high^ EC-lined vessels (tdTomato^+^GFP^high^, i.e., arterioles) in the tibias from wt and EC miR-126 KO (*miR-126^ECΔ/Δ^*) tamoxifen-treated Tie2-CreER/TdTomato/Tg(Ly6a-GFP) reporter mice (n=3 mice per group). **g,** Representative plots (top) and aggregate results (bottom) of BM Sca-1^high^ (tdTomato^+^GFP^high^) and Sca-1^low^ (tdTomato^+^GFP^low^) EC subfractions from the same mice as in **f**. **h,** Calvarial vessel permeability as shown by dextran (labelled with TRITC,150kDa, PE) leakiness in wt and miR-126^ECΔ/Δ^ mice assessed at 30 min post dextran administration using intravital confocal microscopy. White arrows indicate dextran leakiness observed mainly outside the vessels from miR-126^ECΔ/Δ^ mice. One of three independent experiments with similar results is shown. Results represent mean±SEM. Significance values: *, p<0.05; **, p<0.01; ***, p<0.001.

**Figure S10. TNFα-miR-126 axis mediates vascular remodeling of the leukemic BM niche. a-b,** Representative plots (**a**) and aggregate results (**b**) of CD31^+^Sca-1^low^ subfractions in BM ECs from normal wt mice exposed *in vitro* to vehicle, mrTNFα (1ng/ml), or mrTNFα + miR-126 mimic (2µM) for 48 hrs, analyzed by flow cytometry analysis (n=4 mice per group). **c,** miR-126 levels in BM ECs from normal wt mice exposed *in vitro* to vehicle, mrTNFα (1ng/ml), mrTNFα+anti-TNFαR1 (1µg/ml), mrTNFα+anti-TNFαR2 (1µg/ml), or mrTNFα+anti-TNFαR1+anti-TNFαR2 for 24 hrs, as analyzed by Q-RT-PCR (n=6). **d-e,** Quantification of CD31^+^Sca-1^high^ EC-lined vessels (i.e., arterioles) in tibias (**d**, n=3 mice per group), and BM EC Sca-1^high^ and Sca-1^low^ subfractions by flow cytometry analysis (**e**, n=4 mice per group), from normal wt mice treated *in vivo* with vehicle, mrTNFα (1µg/day, ip) or mrTNFα+miR-126 mimics (20mg/kg/day, iv) for 3 wks. **f,** miR-126 levels in BM ECs from wt recipient mice engrafted with BM MNCs from normal wt or *Mll*^PTD/WT^/*Flt3*^ITD/ITD^ AML donors (n=5 mice per group). **g,** miR-126 levels in BM ECs from AML mice treated with IgG or TNFα-neutralizing antibody (anti-TNFα, 1mg, ip, 4 times/wk, 3 wks; n=5 mice per group) analyzed by Q-RT-PCR. **h-I,** CD31 (FITC) and Sca-1 (PE) IF staining (**h**, left) and quantification (**h**, right) of CD31^+^Sca-1^high^ EC-lined vessels (i.e., arterioles) in tibias, and representative plots (**i**, left) and aggregate results (**i**, right) of BM EC Sca-1^high^ and Sca-1^low^ subfractions, from wt recipient mice receiving BM MNCs from *Mll*^PTD/WT^/*Flt3*^ITD/ITD^ AML donors (AML to wt, 2x10^6^/mouse, n=3 mice per group). Yellow arrows indicate CD31^+^Sca-1^high^ EC-lined vessels; white arrows indicate CD31^+^Sca-1^low^ EC-lined vessels; scale bars represent a size of 100µm. **j,** miR-126 levels in BM ECs from NSGS mice engrafted with CB CD34^+^ cells or human primary FLT3-ITD+ AML blasts (n=5 mice per group), analyzed by Q-RT-PCR. **k-l,** CD31 (FITC) and Sca-1 (PE) IF staining (**k**, left) and quantification (**k**, right) of CD31^+^Sca-1^high^ EC-lined vessels (i.e., arterioles) in tibias, and representative plots (**l**, left) and aggregate results (**l**, right) of BM EC Sca-1^high^ and Sca-1^low^ subfractions, from *Spred1^ECΔ/Δ^* mice (EC miR-126 OE) treated *in vivo* with mrTNFα (1µg/day, ip, 3 wks) or vehicle, analyzed by flow cytometry analysis (n=3 mice per group). Yellow arrows indicate CD31^+^Sca-1^high^ EC-lined vessels; white arrows indicate CD31^+^Sca-1^low^ EC-lined vessels; scale bars represent a size of 100µm. **m,** Quantification of CD31^+^Sca-1^high^ EC-lined vessels (i.e., arterioles) in tibias from wt recipient mice engrafted with normal wt or AML cells, and in tibias from *Spred1^ECΔ/Δ^* recipient mice engrafted with AML cells (n=3 mice per group). Results represent mean±SEM. Significance values: *, p<0.05; **, p<0.01; ***, p<0.001.

**Figure S11. Gata2 mediates TNFα-induced miR-126 downregulation in ECs**. **a,** Gata2 levels in BM ECs from normal and AML mice, analyzed by Q-RT-PCR (n=4). **b-c,** Gata2 (**b**) and miR-126 (**c**) levels in HUVECs transduced with Gata2 siRNA or control siRNA, measured by Q-RT-PCR (n=3). **d-e,** Gata2 mRNA in murine BM ECs from normal wt mice post treatment with mrTNFα (1ng/ml) for 8 hrs (**d**) and in HUVECs treated with human recombinant (hr) TNFα (1ng/ml) for 4 and 16 hrs (**e**), measured by Q-RT-PCR (n=3). **f,** GATA2 protein levels in HUVECs treated with hrTNFα or vehicle for 16 hrs, analyzed by western blot. One of three independent experiments with similar results was shown. **g,** Enrichment of GATA2 on the EGFL7/miR-126 promoter in HUVECs was analyzed by chromatin immunoprecipitation (ChIP) assay (n=3). Chromatin proteins were cross-linked to chromatin with formaldehyde and sheared into 400 – 1000 bp fragments. Nucleoprotein complexes were immunoprecipitated using anti-GATA2 or control IgG antibody. The precipitated DNA fractions were analyzed by quantitative-PCR using primers for the miR-126 promotor region encompassing Gata2. Input DNA was used as a positive control. **h,** Enrichment of GATA2 on the EGFL7/miR-126 promoter in HUVECs treated with hrTNFα (10ng/ml) or vehicle for 24 hrs, analyzed by CHIP assay followed by PCR analysis using primers for the miR-126 promotor region. Results represent mean±SEM. Significance values: *, p<0.05; **, p<0.01; ***, p<0.001; ****, p<0.0001.

**Figure S12. Preventing treatment-induced BM vascular changes by maintaining BM TNFα levels rescues LSC treatment-resistance. a,** Gata2 levels in BM LSKs from AML mice treated with AC220 or vehicle for 3 wks, analyzed by Q-RT-PCR (n=3 mice per groups). **b,** Representative plots of flow cytometry analysis showing gating strategy of AML LSCs (CD45.1/CD45.2 AML LSK) from the BM of AML mice treated with AC220 or vehicle for 3 wks, relative to Fig. 5**g**, **h**. **c,** Representative plots of cell cycling of AML LSKs from AML mice treated with AC220 or vehicle for 3 wks, analyzed by Hoechst and Pyronin Y staining and flow cytometry analysis. One of three independent experiments with similar results was shown. **d,** Representative plots showing gating strategy of BM immunophenotypically defined subpopulations from the diseased *Mll*^PTD/WT^/*Flt3*^ITD/ITD^ AML mice analyzed by flow cytometry (left) and frequency of leukemia-initiating cells (LICs) in these subpopulations (CD45.2) assessed using limiting-dilution transplantation of BM subpopulations into congenic CD45.1 recipient mice (right; 100, 500, 2000 and 10000 cells/mouse, n=6-15 mice per group). **e,** WBC counts and AML donor cell burden in the PB of recipient mice receiving BM cells from AC220-treated or vehicle-treated AML donors (1x10^6^/mouse) (n=10 mice per group). **f,** Levels of miR-126 in BM ECs from AML mice treated with AC220 (20mg/kg/day, oral gavage) + mrTNFα (1µg/day, iv) or AC220 + vehicle for 3 wks, analysed by Q-RT-PCR (n=5 mice per group). **g,** Schematic design of the experiments. Human *FLT3-ITD*+ AML blasts (2x10^6^/mouse) were transplanted into NSGS mice (3Gy) to generate patient-derived xenograft (PDX) model. Upon detection of AML engraftment at 4 wks after transplantation (i.e., 20-40% of circulating human CD45^+^ cells), the mice were divided into two groups and treated with AC220 (20mg/kg/day, oral gavage) + mrTNFα (1µg/day, iv) or AC220 + vehicle for 3 wks. A cohort of treated mice were monitored for survival (n=8 mice per group). Another cohort of mice were euthanized, vascular distribution (n=4 mice per group) and leukemic burden (n=9 mice per group) were analyzed, and BM cells were transplanted into 2^nd^ recipient mice for evaluation of post-treatment LSC burden. **h-i,** Long bones (femurs and tibias) from AML PDX mice treated with AC220+mrTNFα or AC220+vehicle for 3 wks were evaluated for: BM EC Sca-1^high^ and Sca-1^low^ subfractions by flow cytometry analysis (**h**, left, representative plots; right, aggregate results) and tibial CD31^+^Sca-1^high^ EC-lined vessels (i.e., arterioles) by CD31 (FITC) and Sca-1 (PE) IF staining (**i**, left) and quantification (**i**, right) (n=4 mice per group). Yellow arrows indicate CD31+Sca-1^high^ EC-lined vessels; white arrows indicate CD31+Sca-1^low^ EC-lined vessels; scale bars represent a size of 50µm. **j,** Frequency of human CD45^+^CD34^+^ LSCs in the BM from AC220+mrTNFα-treated and AC220+vehicle-treated PDX mice, as analyzed by flow cytometry (n=9 mice per group). **k,** Survival of primary treated PDX mice (left, n=8 mice per group) and 2^nd^ recipients **(right, n=10 mice per group) receiving BM cells from AC220+**mrTNFα-treated or **AC220+vehicle**-treated PDX donors. Results represent mean±SEM. Significance values: *, p<0.05; **, p<0.01; ***, p<0.001; ****, p<0.0001; ns, not significant.

**Figure S13. BM vascularization in AC220-treated miR-126^ECΔ/Δ^ versus AC220-treated miR-126 wt leukemic mice. a-b,** Representative plots (**a**) and aggregate results (**b**) of Sca-1^high^ and Sca-1^low^ subfractions in BM CD45^-^Ter119^-^CD31^+^ ECs from wt and miR-126^ECΔ/Δ^ leukemic mice treated with AC220 for 3 wks as shown in Fig. 6**F**, analysed by flow cytometry (n=4 mice per group). **c-d,** CD31 (FITC) and Sca-1 (PE) IF staining (**c**) and quantification (**d**) of CD31^+^Sca-1^high^ EC-lined vessels (i.e., arterioles) in the tibias from wt and miR-126^ECΔ/Δ^ leukemic mice treated with AC220 for 3 wks as shown in Fig. 6**f**. For **c**: yellow arrows indicate CD31^+^Sca-1^high^ EC-lined vessels (i.e., arteriole); white arrows indicate CD31^+^Sca-1^low^ EC-lined vessels (i.e., sinusoid). Results represent mean±SEM. Significance values: *, p<0.05; **, p<0.01.

**Figure S14. Preventing treatment-induced BM vascular changes by miRisten rescues LSC treatment-resistance in AML murine model. a,** Schematic design of the experiments. BM MNCs from diseased *Mll*^PTD/wt^/*Flt3*^ITD/ITD^ AML mice (CD45.1/CD45.2) were transplanted into normal wt recipient mice (CD45.1, 6Gy). Starting at day 14 post transplantation, mice were treated with SCR, miRisten (20mg/kg/day, iv), SCR + AC220 (20mg/kg/day, oral gavage), or miRisten + AC220 for 3 wks. **b-f,** After completion of treatment, the mice treated with SCR, miRisten, SCR+**AC220, or miRisten+** **AC220** were evaluated for: miR-126 levels in ECs analyzed by Q-RT-PCR (**b**, n=4 mice per group), WBC counts, AML cell burden in PB, BM and spleen (**c**, n=8 mice per group), spleen size (**d**), BM LSCs (AML LSKs) (**e**, n=8 mice per group), and survival (**f**, n=7 to 10 mice per group)**. g, WBC counts and AML engraftment rates and survival in** 2^nd^ recipient mice **receiving BM cells from** AML donors treated with SCR, miRisten, SCR+**AC220, or miRisten+AC220** **for 3 wks (n=10 mice per group)**. Results represent mean±SEM. Significance values: *, p<0.05; **, p<0.01; ***, p<0.001; ****, p<0.0001.

**Figure S15. Preventing treatment-induced BM vascular changes by miRisten rescues LSC treatment-resistance in AML PDX model. a,** Schematic design of the experiments. NSGS mice were transplanted with human FLT3-ITD+ AML MNCs. Starting at day 30 post transplantation, mice were treated with miRisten (20mg/kg/day, iv) + AC220 (20mg/kg/day, oral gavage) or SCR + AC220 for 3 wks. **b,** Representative plots (left) and aggregate results (right) of human CD45^+^CD33^+^ AML cell engraftment in the BM of leukemic NSGS mice treated with miRisten + AC220 or SCR + AC220 for 3 wks, analyzed by flow cytometry (n=10 mice per group). **c,** **Survival of primary treated leukemic mice (left, n=11 mice for miRisten+AC220 and n=13 mice for SCR+AC220) and** 2^nd^ recipients **(right, n=8 mice per group) receiving BM cells from** miRisten+A**C220**-treated or **SCR+AC220**-treated PDX donors. Results represent mean±SEM. Significance values: ***, p<0.001.

**Table S1. Information of AML patient samples used in this study**

| **Sample** | **Source** | **Disease status** | Risk status | Status | WBC (K) | Blasts % |
| --- | --- | --- | --- | --- | --- | --- |
| AML 866 | BM | untreated | Poor-risk | Normal Cytogenetic, FLT3-ITD positive, FLT3 D835 negative, JAK2 negative | 86.3 | 90 |
| AML 1898 | BM | untreated | Poor-risk | Rising from MDS, Abnormal cytogenetics with trisomy 8, FLT3-ITD positive, FLT3-TKD negative, NPM1 negative, CEBOA negative, IDH1 negative, IDH2 negative | 10 | 51 |
| AML 1940 | PB | untreated | Poor-risk | Normal karyotype, FLT3-ITD positive, FLT3-TKD negative, IDH1 negative, IDH2 negative, CEBPA positive, NPM1 negative | 97 | 53 |
| AML 1959 | PB | Induction failure | Poor-risk | 46, XY, t(2;17)(q23;q21), der(8)t(8;8) (p23;q13) [4]/[22], 8% gain of MYC by FISH, FLT3-ITD positive, FLT3-TKD negative | 62.3 | 88 |
| AML 2240 | PB | Relapsed | Poor-risk | Normal karyotype, 1.9% gain of MYC, FLT3-ITD positive, FLT3-TKD negative | 65.9 | 52 |
| AML 2224 | PB | Relapsed | Poor-risk | Normal Cytogenetics, FLT3-ITD positive, FLT3-TKD negative, NPM1 positive, IDH1 negative, IDH2 negative, MPL negative | 24.3 | 18 |
| AML 2360 | BM | untreated | Poor-risk | Normal Cytogenetics, FLT3-ITD positive, FLT3-TKD negative, NPM1 negative, C-KIT negative, CEBPA negative, IDH1 negative, IDH2 negative | 21.3 | 52 |
| AML 2431 | PB | untreated | Poor-risk | Abnormal cytogenetics with del(9), FLT3-ITD positive, FLT3-TKD negative, CEBPA negative, NPM1 negative, IDH1 negative, IDH2 negative | 62 | 90 |
| AML 2514 | BM | untreated | Poor-risk | Normal Cytogenetics, FLT3-ITD positive, FLT3-TKD negative, CEBPA negative, NPM1 positive, IDH1 negative, IDH2 negative | 59.4 | 87 |
| AML 2568 | PB | Relapsed | Poor-risk | Normal cytogenetics, FLT3-ITD positive, FLT3-TKD negative, NPM1 positive, CEBPA negative, IDH1 negative, IDH2 negative | 38.3 | 57 |

**Table S2.** **Gene ontology analysis of differentially expressed genes in Sca-1^high^ ECs versus Sca-1^low^ ECs selected from normal wt mice**

| **Upregulated in Sca-1^high^ ECs vs Sca-1^low^ ECs** | | |
| --- | --- | --- |
| **Term** | P-value | **Adjusted P-value** |
| regulation of angiogenesis (GO:0045765) | 4.4816E-12 | 2.2869E-08 |
| regulation of cell migration (GO:0030334) | 4.9053E-08 | 0.00012516 |
| sprouting angiogenesis (GO:0002040) | 2.5759E-07 | 0.00043815 |
| synapse assembly (GO:0007416) | 2.9352E-07 | 0.00037445 |
| regulation of vasculature development (GO:1901342) | 3.4252E-07 | 0.00034957 |
| regulation of endothelial cell proliferation (GO:0001936) | 3.7843E-07 | 0.00032186 |
| positive regulation of cell differentiation (GO:0045597) | 8.4503E-07 | 0.00061603 |
| ventricular septum morphogenesis (GO:0060412) | 2.286E-06 | 0.0014582 |
| positive regulation of cell migration (GO:0030335) | 2.4576E-06 | 0.00139346 |
| positive regulation of vasculature development (GO:1904018) | 2.7912E-06 | 0.00142434 |
| negative regulation of sprouting angiogenesis (GO:1903671) | 4.1349E-06 | 0.00191821 |
| Notch signaling involved in heart development (GO:0061314) | 4.307E-06 | 0.00183154 |
| regulation of vasculogenesis (GO:2001212) | 5.1103E-06 | 0.00200598 |
| negative regulation of cell differentiation (GO:0045596) | 5.7304E-06 | 0.00208872 |
| artery morphogenesis (GO:0048844) | 6.4484E-06 | 0.00219376 |
| negative regulation of angiogenesis (GO:0016525) | 6.5576E-06 | 0.00209147 |
| cellular response to growth factor stimulus (GO:0071363) | 7.1672E-06 | 0.00215142 |
| positive regulation of angiogenesis (GO:0045766) | 7.2124E-06 | 0.00204473 |
| negative regulation of blood vessel endothelial cell migration (GO:0043537) | 9.8124E-06 | 0.0026354 |
| blood circulation (GO:0008015) | 1.0375E-05 | 0.00264709 |
| cell-cell adhesion via plasma-membrane adhesion molecules (GO:0098742) | 1.1552E-05 | 0.00280712 |
| positive regulation of MAPK cascade (GO:0043410) | 1.2055E-05 | 0.00279626 |
| vasculogenesis (GO:0001570) | 1.4629E-05 | 0.00324572 |
| ventricular septum development (GO:0003281) | 1.729E-05 | 0.00367634 |
| enzyme linked receptor protein signaling pathway (GO:0007167) | 1.7951E-05 | 0.0036642 |
| regulation of endothelial cell migration (GO:0010594) | 2.404E-05 | 0.00471823 |
| cell surface receptor signaling pathway involved in heart development (GO:0061311) | 2.5504E-05 | 0.00482027 |
| regulation of Notch signaling pathway (GO:0008593) | 3.0176E-05 | 0.00549952 |
| negative regulation of endothelial cell migration (GO:0010596) | 3.077E-05 | 0.00541438 |
| blood vessel endothelial cell migration (GO:0043534) | 3.2069E-05 | 0.0054549 |
| extracellular matrix organization (GO:0030198) | 3.2854E-05 | 0.0054082 |
| negative regulation of blood vessel morphogenesis (GO:2000181) | 3.3959E-05 | 0.00541534 |
| positive regulation of actin filament bundle assembly (GO:0032233) | 3.4117E-05 | 0.00527567 |
| regulation of anatomical structure morphogenesis (GO:0022603) | 3.4899E-05 | 0.00523794 |
| regulation of MAPK cascade (GO:0043408) | 3.8054E-05 | 0.00554822 |
| hippo signaling (GO:0035329) | 3.8437E-05 | 0.00544842 |
| negative regulation of epithelial cell proliferation (GO:0050680) | 4.2752E-05 | 0.00589624 |
| positive regulation of osteoblast differentiation (GO:0045669) | 4.3526E-05 | 0.00584503 |
| positive regulation of transcription from RNA polymerase II promoter (GO:0045944) | 4.6755E-05 | 0.00611777 |
| atrioventricular valve morphogenesis (GO:0003181) | 4.9579E-05 | 0.00632506 |
| positive regulation of endothelial cell proliferation (GO:0001938) | 5.3512E-05 | 0.00666029 |
| negative regulation of cell migration (GO:0030336) | 5.7245E-05 | 0.00695528 |
| calcium-dependent cell-cell adhesion via plasma membrane cell adhesion molecules (GO:0016339) | 6.2022E-05 | 0.00736047 |
| pulmonary valve morphogenesis (GO:0003184) | 7.101E-05 | 0.00823549 |
| vascular smooth muscle cell development (GO:0097084) | 7.1564E-05 | 0.00811536 |
| positive regulation of stress fiber assembly (GO:0051496) | 8.3325E-05 | 0.0092436 |
| mitral valve morphogenesis (GO:0003183) | 8.7189E-05 | 0.00946653 |
| artery development (GO:0060840) | 8.9968E-05 | 0.00956471 |
| transmembrane receptor protein serine/threonine kinase signaling pathway (GO:0007178) | 9.875E-05 | 0.01028416 |
| regulation of signal transduction (GO:0009966) | 0.00010557 | 0.01077444 |
| negative regulation of signal transduction (GO:0009968) | 0.00010708 | 0.01071414 |
| positive regulation of cytokine-mediated signaling pathway (GO:0001961) | 0.00011046 | 0.01084034 |
| positive regulation of intracellular signal transduction (GO:1902533) | 0.0001243 | 0.01196827 |
| Notch signaling pathway (GO:0007219) | 0.00015318 | 0.0144752 |
| cellular response to vascular endothelial growth factor stimulus (GO:0035924) | 0.00018211 | 0.01689677 |
| chloride ion homeostasis (GO:0055064) | 0.00019842 | 0.01808072 |
| pulmonary valve development (GO:0003177) | 0.00019842 | 0.01776351 |
| regulation of cell migration involved in sprouting angiogenesis (GO:0090049) | 0.00020168 | 0.01774399 |
| negative regulation of endothelial cell proliferation (GO:0001937) | 0.00020168 | 0.01744325 |
| actin filament bundle organization (GO:0061572) | 0.0002063 | 0.0175461 |
| endothelial cell migration (GO:0043542) | 0.0002063 | 0.01725845 |
| response to molecule of bacterial origin (GO:0002237) | 0.00020955 | 0.01724734 |
| mitral valve development (GO:0003174) | 0.00022854 | 0.01851179 |
| dorsal aorta morphogenesis (GO:0035912) | 0.00022854 | 0.01822254 |
| dorsal aorta development (GO:0035907) | 0.00022854 | 0.0179422 |
| positive regulation of protein phosphorylation (GO:0001934) | 0.00023986 | 0.01854533 |
| heart trabecula morphogenesis (GO:0061384) | 0.00025192 | 0.01918763 |
| negative regulation of cell migration involved in sprouting angiogenesis (GO:0090051) | 0.00025409 | 0.01906784 |
| regulation of osteoblast differentiation (GO:0045667) | 0.00026433 | 0.01954873 |
| positive regulation of cell motility (GO:2000147) | 0.00026532 | 0.019342 |
| positive regulation of ossification (GO:0045778) | 0.00027383 | 0.0196812 |
| regulation of axon extension involved in axon guidance (GO:0048841) | 0.00032988 | 0.02338049 |
| actin filament bundle assembly (GO:0051017) | 0.00036525 | 0.02553222 |
| cell junction assembly (GO:0034329) | 0.00036597 | 0.02523691 |
| negative regulation of cell-matrix adhesion (GO:0001953) | 0.00036923 | 0.02512265 |
| negative regulation of peptidyl-tyrosine phosphorylation (GO:0050732) | 0.00036923 | 0.02479209 |
| endothelial cell development (GO:0001885) | 0.00039498 | 0.02617671 |
| endocardial cushion development (GO:0003197) | 0.00039498 | 0.02584111 |
| cell-matrix adhesion (GO:0007160) | 0.00042724 | 0.02759741 |
| negative regulation of coagulation (GO:0050819) | 0.00043304 | 0.02762223 |
| monovalent inorganic anion homeostasis (GO:0055083) | 0.00043304 | 0.02728121 |
| regulation of stress fiber assembly (GO:0051492) | 0.00044808 | 0.02788455 |
| positive regulation of peptidyl-tyrosine phosphorylation (GO:0050731) | 0.00045034 | 0.02768761 |
| negative regulation of cell communication (GO:0010648) | 0.00045495 | 0.02763832 |
| cell-cell junction organization (GO:0045216) | 0.00046084 | 0.02766641 |
| heart morphogenesis (GO:0003007) | 0.00060995 | 0.03619268 |
| negative regulation of Notch signaling pathway (GO:0045746) | 0.00069406 | 0.04071011 |
| Notch receptor processing (GO:0007220) | 0.00069987 | 0.04058431 |
| cardiac ventricle development (GO:0003231) | 0.00069987 | 0.04012831 |
| cardiac epithelial to mesenchymal transition (GO:0060317) | 0.00069987 | 0.03968244 |
| aorta morphogenesis (GO:0035909) | 0.00069987 | 0.03924637 |
| cellular response to BMP stimulus (GO:0071773) | 0.00071298 | 0.03954742 |
| positive regulation of epithelial cell proliferation (GO:0050679) | 0.00078427 | 0.04303379 |
| negative regulation of response to stimulus (GO:0048585) | 0.00078901 | 0.04283291 |
| negative regulation of epithelial cell migration (GO:0010633) | 0.00087065 | 0.04676754 |
| establishment of endothelial barrier (GO:0061028) | 0.0008766 | 0.0465967 |
| cellular response to cytokine stimulus (GO:0071345) | 0.00092326 | 0.04857111 |
| positive regulation of I-kappaB kinase/NF-kappaB signaling (GO:0043123) | 0.00094496 | 0.04920562 |
| **Downregulated in Sca-1^high^ ECs vs Sca-1^low^ ECs** | | |
| **Term** | **P-value** | **Adjusted P-value** |
| platelet aggregation (GO:0070527) | 3.2286E-07 | 0.00164757 |
| cell projection assembly (GO:0030031) | 3.2464E-07 | 0.00082831 |
| regulation of small GTPase mediated signal transduction (GO:0051056) | 1.4872E-06 | 0.00252976 |
| homotypic cell-cell adhesion (GO:0034109) | 3.1118E-06 | 0.00396994 |
| platelet degranulation (GO:0002576) | 4.6639E-06 | 0.00475997 |
| microtubule cytoskeleton organization involved in mitosis (GO:1902850) | 5.8923E-06 | 0.00501136 |
| regulated exocytosis (GO:0045055) | 6.0572E-06 | 0.00441568 |
| peptidyl-tyrosine autophosphorylation (GO:0038083) | 3.673E-05 | 0.02342935 |
| centromere complex assembly (GO:0034508) | 3.8212E-05 | 0.02166612 |
| regulation of B cell receptor signaling pathway (GO:0050855) | 4.0599E-05 | 0.02071785 |
| regulation of platelet aggregation (GO:0090330) | 5.4064E-05 | 0.02508099 |
| integrin-mediated signaling pathway (GO:0007229) | 8.9097E-05 | 0.03788856 |
| peptidyl-tyrosine phosphorylation (GO:0018108) | 8.9221E-05 | 0.03502283 |
| regulation of mast cell degranulation (GO:0043304) | 0.00010395 | 0.0378904 |
| lipoxin biosynthetic process (GO:2001301) | 0.00011805 | 0.04015913 |
| regulation of platelet activation (GO:0010543) | 0.00012391 | 0.03951965 |
| phosphorylation (GO:0016310) | 0.00013135 | 0.03942949 |
| kinetochore organization (GO:0051383) | 0.00013753 | 0.03898849 |
| regulation of antigen receptor-mediated signaling pathway (GO:0050854) | 0.00013753 | 0.03693646 |
| carboxylic acid transport (GO:0046942) | 0.00019377 | 0.04943992 |

**Table S3.** **Taqman gene assay list**

| **Gene name** | **Assay ID** |
| --- | --- |
| Ang(angiogenin) | Mm00833184_s1 |
| Angpt1 (angiopoietin-1, Ang1) | Mm00456503_m1 |
| Angpt2 (angiopoietin-2, Ang2) | Mm00545822_m1 |
| Angptl2 (angiopoietin-like 2) | Mm00507897_m1 |
| Angptl3 (angiopoietin-like 3) | Mm00803820_m1 |
| Igf1 | Mm00439560_m1 |
| Igfbp2 | Mm00492632_m1 |
| Hgf | Mm01135184_m1 |
| Hif1a | Mm00468869_m1 |
| Vegfa | Mm00437306_m1 |
| Vegfb | Mm00442102_m1 |
| Vegfc | Mm00437310_m1 |
| Figf (Vegfd) | Mm01131929_m1 |
| Spred1 | Mm01277511_m1 |
| Vcam1 | Mm01320970_m1 |
| Pik3r2 | Mm00435694_m1 |
| Adam9 | Mm01218460_m1 |
| Cdk3 (Cdk3-ps) | Mm00518387_m1 |
| Vegfr1 (Flt1) | Mm00438980_m1 |
| Kdr (Vegfr2, Flk1) | Mm01222421_m1 |
| Flt4 (Vegfr3) | Mm01292604_m1 |
| Tnfr1 (Tnfrsf1a) | Mm00441883_g1 |
| Tnfr2 (Tnfrs1b) | Mm00441889_m1 |
| Ets1 | Mm01175819_m1 |
| Ets2 | Mm00468973_m1 |
| Gata2 | Mm00492301_m1 |
| Egfl7 | Mm00618004_m1 |
| Cxcl12 | Mm00445553_m1 |
| Kitl (SCF) | Mm00442972_m1 |
| Fgf1 | Mm00438906_m1 |
| Fgf2 | Mm01285715_m1 |
| Fgfr1 | Mm00438930_m1 |
| Fgfr2 | Mm01269930_m1 |
| Cxcr4 | Mm01996749_s1 |
| Pecam-1 (CD31) | Mm01242576_m1 |
| Cdh5 (VE-cadherin) | Mm00486938_m1 |
| Ly6a (Sca-1) | Mm00726565_s1 |
| Endomucin (Emcn) | Mm00497495_m1 |
| Lepr | Mm00440181_m1 |
| Nestin | Mm00450205_m1 |
| E-selectin (selectin, Sele) | Mm00441278_m1 |
| P-selectin (selectin, Selplg) | Mm01204601_m1 |
| claudin 5-Cldn5 | Mm00727012_s1 |
| Tjp1-tight junction protein 1, ZO1 | Mm00493699_m1 |
| Stab1 (stabilin 1) | Mm00460390_m1 |
| Bcl-2 | Mm00477631_m1 |
| β2M | Mm00437762_m1 |
| Hprt1 | Mm03024075_m1 |
| miR-126 | 2228 |
| RNU44 | 111094 |
| RNU48 | 1006 |
| snoRNA234 | 1234 |
| pri/pre-miR-126a | Mm04335191_s1 |
| Pri/pre-miR-126 | Hs04273250_s1 |
| SPRED1 | Hs01084559_m1 |
| VCAM1 | Hs01003372_m1 |
| BCL-2 | Hs00608023_m1 |
| β2M | Hs00187842_m1 |

**Supplementary materials and methods**

**Immunofluorescent staining and 3D confocal imaging of long bones**

Long bones (tibias or femurs) from the mice were processed, sectioned and imaged as described previously(1) with ad-hoc modifications. Briefly, the bone was dissected from mice and surrounding muscle was removed without causing any mechanical damage to bones. The dissected bone was immediately fixed with 4% paraformaldehyde (PFA) for 4 hrs. Then, the bone was washed three times with PBS at 4^0^C and processed for decalcification. It was incubated in decalcification solution (0.5M EDTA, pH 7.4-7.6) for 24 to 48 hrs, washed three times with PBS and continuously incubated with ice-cold cryoprotectant (CPT) solution at 4^0^C for 24 hrs. After incubating with embedding media (EBM) for 45 min at 60^0^C in a water bath, the bone was embedded in a tissue mold. The bone tissue mold was dried at room temperature (RT) for 30 min and stored at -80^0^C at least over night until sectioning. Tissue sections were cut at -23^0^C using a microtome with a thickness of 40 µm and transferred to a microscope slide for staining. The sections were rehydrated with PBS at RT for 5 min, incubated with permeabilization (PRM) solution for 20 min, and blocked with freshly made blocking solution containing BSA for 30 min. The slides were incubated with fluorescent conjugated Abs (Sca-1-PE, CD31-FITC, α-SMA-PE or FITC, Endomucin-FITC; 1:250 dilution) overnight at 4^0^C. Then, the slides were washed three times with PBS and nuclear staining was performed with 4′,6-diamidino-2-phenylindole (DAPI, BD) mounting solution. Imaging was performed using a confocal microscope (Zeiss, LSM880) and analyzed using the Zen program (Zeiss). We also performed high-resolution 3D confocal imaging using long bones from EC/Sca-1 reporter mice [i.e., tamoxifen-treated Tie2-CreER/TdTomato/Tg(Ly6a-GFP)], which allow us to visualize Sca‑1^high^ EC (i.e., tdTomato^+^GFP^high^) and Sca-1^low^ EC (i.e., tdTomato^+^GFP^low^) lined vessels. To quantify Sca-1^high^ EC-lined arterioles in the images, we used a threshold of 80 as a cutoff for the Sca-1-PE channel. Connected objects were identified and those with fewer than 15 pixels were removed to reduce noise using MATLAB’s bwareafilt function. The image was then morphologically closed with a 1.0-pixel filter using MATLAB’s imclose function to close any holes. The perimeter:area ratio was calculated for each object; objects with a ratio of less than 0.7 were removed using MATLAB’s bwconncomp and bwperim functions. The goal was to identify blood vessels as they are thinner than other objects in the image. Finally, the fractions of pixels (i.e., arbitrary unit) that registered as positive for blood vessels within three representative hand-drawn regions of interest were reported as the quantification of arterioles.

**Intravital Imaging**

Intravital confocal microscopy was used to image the calvarium BM vasculature to study the vascular permeability, as previously described(2). Briefly, mice were anesthetized using intraperitoneal injection of 350-400 μl saline solution containing 120 mg/kg ketamine, 10 mg/kg xylazine, and 3 mg/kg acepromazine (ACE). After confirming proper anesthesia by testing for foot reflex, we applied eye ointment to keep the eyes moist during surgery. Prior to imaging, mice were injected intravenously with fluorescent dextran (i.e., TRITC-, FITC- or Alex647-150kDa dextran) to label the vasculature. We shaved the head, revealed the calvaria by a skin incision and held it in a stereotaxic skull holder. Then image acquisition was performed using confocal microscopy (LSM880, Zeiss).

### Flow cytometry analyses

Human CD34^+^ blasts were selected using indirect CD34 microbead kit (Miltenyi Biotec, San Diego, CA). Mouse cells were obtained from PB, BM (from both tibias and femurs), or spleen. For analysis of stem and progenitor cells, c-kit^+^ cells were selected using anti-mouse CD117 microbeads or Lineage^-^ (Lin^-^) cells were selected using mouse Lineage cell depletion kit (both from Miltenyi Biotec, San Diego, CA). The following mouse antibodies were used: mouse biotinylated lineage markers (Ter-119, CD3, NK1.1, IgM, CD4, CD8a, CD19, Gr-1, CD11b, B220), CD45-PE or FITC or APC-eflu780, Ter119-APC-eflu780, CD31-PE-cy7 or APC, Flt3-biotin or PE, CD117 (ACK2, c-kit)–APC-eFlu780, Sca-1-PE or PE-Cy7, and streptavidin-PE or FITC or PerCP-Cy5.5 (all from eBioscience, San Diego, CA). Stem and progenitor populations were identified as LSK cells (Lin^−^Sca-1^hi^c-Kit^hi^). Endothelial cells (ECs) were identified as CD45^-^Ter119^-^CD31^+^. Sca-1^high^ and Sca-1^low^ EC subfractions from *in vitro* studies and from *in vivo* studies were analyzed. To determine the frequency of quiescent LSCs, cell cycle was analyzed by Ki-67-Alexa Fluor 647 (B56, BD) and DAPI (BD) labeling, or Hoechst and Pyronin Y (Sigma) staining, based on the manufacturer’s protocol. All analyses were performed on a Fortessa x20 flow cytometer (BD Biosciences) and sorting was performed on Aria Fusion or ARIA SORP instruments (BD Biosciences) and data were analyzed by BD FACSDiva or FlowJo software.

**Irradiation and transplantation**

To evaluate the impact of vascular changes on LSC burden, BM MNCs (2x10^6^ cells/mouse) from diseased *Mll*^PTD/WT^/*Flt3*^ITD/ITD^ AML mice (CD45.1/CD45.2, B6) were transplanted into Cre+ or Cre- miR-126^flox/flox^/Tie2-cre, Spred1^flox/flox^/Tie2-cre and tamoxifen-administrated miR-126^flox/flox^/Tie2-CreER/TdTomato/Tg(Ly6a-GFP) reporter mice (all CD45.2). For efficacy studies, a cohort of AML mice with a similar disease onset time were generated by transplanting AML MNCs from diseased *Mll*^PTD/WT^/*Flt3*^ITD/ITD^ AML mice (CD45.2 or CD45.1/CD45.2, B6) into congenic recipient mice (CD45.1 B6, from Charles River, B6-Ly5.1, Kingston, US). Since this study focused on the changes in vascular niches caused by AML cell growth, we tried to limit the impact of irradiation on the microenvironment. To determine the lowest irradiation dose that could ensure a successful AML engraftment while minimizing microenvironment and normal hematopoietic toxicity, we conducted a dose-finding study by irradiating wt mice with distinct dose levels (i.e., 4.5, 6, 7.5 and 9 Gy, X-RAD 320 irradiator) before AML cell transplantation. We found that only a fraction of the recipient mice irradiated with 4.5 Gy developed AML, while all the recipient mice irradiated with 6, 7.5 and 9 Gy developed AML with survival days reversely associated with irradiation dose. Therefore, in order to minimize irradiation exposure, we selected 6 Gy as an optimal dose to sub-lethally irradiate the recipient mice before AML transplant for studies aimed at comparing BM vascular changes. B6 recipient mice used in this study were 6 to 10 wks old male and female mice and were irradiated at 6 Gy (X-RAD 320 irradiator) within 24 hrs before transplantation. NSGS mice were 6 to 10 wks old male and female mice and were irradiated at 3 Gy within 24 hrs before transplantation. The number of mice for each study group was chosen based on the expected endpoint variation (i.e., engraftment rate and latency period of leukemia) and on the availability of mice from different strains. All animals studied were compared within litters and/or age- and sex-matched. Mice of the same gender and age were randomly divided into groups. Investigators were blinded to mouse genotype while performing treatment or monitoring engraftment or survival. Mouse care and experimental procedures were performed in accordance with federal guidelines and protocols and were approved by the Institutional Animal Care and Use Committee at the City of Hope (under the approved protocol IACUC 15005).

**Isolation of endothelial cells (ECs) from long bones**

### Tibias and femurs were excised from the mice. After removing the muscle and connective tissue, the bones were flushed 5 times using a 23-gauge needle and 3 ml of cold Iscove's Modified Dulbecco's Medium (IMDM, ThermoFisher) and collected as central marrow. The marrow-depleted bones were crushed gently with a mortar and pestle in cold IMDM and the bone fragments were incubated at 37 °C with 3ml of 3mg/ml collagenase I (Sigma) and gently agitated for 45 min. The digested bones were then filtered through a 40 μm strainer (BD Bioscience) and collected as endosteal marrow. Central marrow and endosteal marrow were combined and then stained and analyzed by flow cytometry. CD45^-^Ter119^-^CD31^+^ ECs and Sca-1^high^ and Sca-1^low^ subfractions were also sorted by flow cytometry for *in vitro* cultures. Mouse BM ECs were cultured in complete EGM-2 medium (CC-3162, Lonza), at 37°C with 5% CO_2_ and 21% O_2_ and high humidity.

**Lentiviral transduction of murine EC cells**

GFP-expressing miRZip anti-miR-126 (miR-126 KD), miR-126 precursor (miR-126 OE) and control lentivirus (System Biosciences) supernatants were produced and used for transduction of murine CD45^-^Ter119^-^CD31^+^ ECs. Briefly, Murine BM ECs were exposed to miR-126 KD, miR-126 OE or control lentiviral supernatant [multiplicity of infection (MOI) = 10] supplemented with 1xTransDux virus transduction reagent (System Biosciences), and GFP^+^ cells were sorted at 48 h after transduction.

**Measurement of cytokine and chemokine levels in mouse blood and marrow by Luminex Assay**

BM cells were flushed 5 times from the femurs of AML or wt mice using 200 µL IMDM/mouse. BM cell suspension was centrifuged, and supernatants were collected. 0.5ml peripheral blood per mouse was collected and plasma was obtained. Luminex xMAP technology for a panel of murine cytokines and chemokines (MILLIPLEX MAP Mouse Cytokine/Chemokine Panel: Eotaxin/CCL11, G-CSF, GM-CSF, IFN-γ, IL-1α, IL-1β, IL-2, IL-3, IL-4, IL-5, IL-6, IL-7, IL-9, IL-10, IL-12 (p40), IL-12 (p70), IL-13, IL-15, IL-17, IP-10, KC, LIF, LIX, MCP-1, M-CSF, MIG, MIP-1α, MIP-1β, MIP-2, RANTES, TNF-α, VEGFα; Millipore, MCYTOMAG-70K) were performed with blood plasma and BM supernatants and concentrations calculated using standard curves. The volume of the BM cavities of two femurs is approximately 20 µL, and the measured concentration of cytokines was adjusted based on this dilution factor (200/20= 10x) to obtain the concentration of cytokines per unit volume BM.

**Measurement of TNFα level in BM by immunohistochemistry (IHC)**

BM slides from NSGS mice engrafted with CB CD34+ cells or with human FLT3-ITD+ AML blasts (engraftment rates > 70%) were deparaffinized and immunostained. Briefly, the slides were incubated with anti-TNFα antibody (ab6671, abcam) overnight at 4°C. Slides incubated with IgG were used as the negative controls. Slides were developed using the HRP-DAB kit (R&D Systems) according to the manufacturer's instructions and counterstained with hematoxylin (Sigma, St. Louis, MO).

**RNA sequencing**

Total RNA was extracted from CD45^-^Ter119^-^CD31^+^Sca-1^high^ and CD45^-^Ter119^-^CD31^+^Sca-1^low^ cells sorted from the BM of normal wt mice using the miRNeasy micro Kit (Qiagen, Valencia, CA). SMART-Seq® Ultra Low Input RNA Kit for Sequencing – v4 (TaKaRa, Cat 634888) was used for generating amplified double stranded (ds) cDNA from each sample with 2ng of input total RNA according to the manufacturer's protocol. The resulting ds cDNA was sheared with Covaris LE220 with the setting of DNA fragment size of 200bp peak. The sheared DNA was used for sequencing library preparation by using KAPA HyperPrep Kits (Roche, Cat KK8500). The final libraries were quantified with qubit and bioanalyzer. The sequencing was performed with the single read mode of 51 cycles of read1 and 7 cycles of index read with V4 reagents on Illumina Hiseq2500. Real-time analysis (RTA) 2.2.38 software was used to process the image analysis and base calling. The nf-core RNAseq pipeline v1.3 was used to process the raw sequencing reads (<https://nf-co.re>). Reads were mapped to the mouse genome version GRCm38 using the STAR aligner(3). Quality control was performed using the tools and metrics from the nf-core RNAseq pipeline (<https://nf-co.re>). In brief, the quality control reports showed that each sample was successful and that there were low levels of variance among the samples. The Subread function “featureCounts” was used to produce read counts for each gene(4). DESeq2 was used to quantify expression by normalizing gene counts and to determine differential expression between Sca-1^high^ and Sca-1^low^ samples(5). Expression profiling of a selected panel of genes analyzed by RNA sequencing or by Q-RT-PCR using Fluidigm Biomark (see Gene expression by Q-RT-PCR for details), was compared and presented with heatmaps, shown as log2-transformed ratio using 2^–ΔCt^ method (ΔCt=Ct of target –Ct of *β*2m).

**Chromatin immunoprecipitation (ChIP)**

GATA2 binding to DNA sequence upstream of EGFL7/miR-126 was analyzed by ChIP assay using the MAGnify ChIP system (ThermoFisher) according to the manufacturer’s protocol. In brief, HUVECs (P4 generation) were crosslinked and sheared to fragments by sonification to ~500-2000 bp. Magnetic beads were incubated with 5 μg control IgG or GATA2 IgG (abcam #22849, rabbit) for 1 hr at room temperature before lysate was incubated with bead-antibody complex for 1 hr at 4°C. After washing and reverse crosslinking, DNA was isolated and underwent PCR-based analysis with primers for EGFL7/miR-126 (forward: 5´-ATATAGATCTCAGGCCCCCTCAC ACGGTGG-3´, reverse: 5´-ATATAAGCTTCCACCTGCCTGGCCTGTCCA-3´), along with input as positive control.

**Q-RT-PCR**

To measure the miRNA and mRNA expression, total RNA was extracted using the miRNeasy Mini or micro Kit (Qiagen, Valencia, CA). For miRNA expression, reverse transcription using MultiScribe™ Reverse Transcriptase and Q-PCR analysis using Taqman assays (Applied Biosystems) (Table S3) were performed according to the manufacturer’s protocol. RNU44 and snoRNA234 was used as internal controls for human and mouse miRNA respectively. For mRNA expression in ECs by Fluidigm Biomark, first-strand cDNA was synthesized from 200 CD45^-^Ter119^-^CD31^+^Sca-1^high^ or CD45^-^Ter119^-^CD31^+^Sca-1^low^ cells using the SuperScript III First-Strand Kit and preamplified for 18 cycles, and Q-RT-PCR analysis was performed by multiplex Q-RT-PCR using TaqMan Gene Expression Assays (Applied Biosystems) (Table S3) and the Biomark HD microfluidic system (Fluidigm). For mRNA expression in other cells, first-strand cDNA was synthesized using the SuperScript III First-Strand Kit and then quantitative real-time PCR (QPCR) was performed. Results are presented as log2-transformed ratio according to the 2^–ΔCt^ method (ΔCt=Ct of target –Ct of *β*2m).

**miRNA labeling**

CD45^-^Ter119^-^CD31^+^Sca-1^high^ and CD45^-^Ter119^-^CD31^+^Sca-1^low^ cells sorted from the wt mice, or CD45^-^Ter119^-^CD31^+^ ECs selected from the normal wt mice and treated *in vitro* with mrTNFα (1ng/ml) or vehicle for 24 hrs, were incubated with miR126 SmartFlare RNA probe (EMD Millipore) for 16h. Cells were then washed in 1× phosphate buffered saline (PBS) and fixed in 4% PFA for 3 min. Nuclei were counterstained with DAPI and the images were analyzed using a confocal microscope (Carl Zeiss).

**Oligonucleotide design and synthesis**

miRisten (CpG-miR-126 inhibitor) and CpG-scramble RNA (SCR, control) were designed and synthesized as previously described(6). To make the miR-126 mimic oligonucleotide, miR-126 mimic or scramble RNA (scrRNA, control) was linked with the partially phosphorothioated oligodeoxyribonucleotide (ODN) using 5 units of C3 carbon chain linker, (CH2)3 (indicated by x), and generated the miR-126 mimic passenger. The guide and passenger oligonucleotides were then mixed and heated to 80°C for 1 min, followed by incubating at 37°C for 1 h, to generate miR-126 mimics. The sequences were as follows: **miR-126-3p-mimic (guide)**: 5'- rUrCrGrUrArCrCrGrUrGrArGrUrArArUrArArUrGrCrG-3'; **CpG-miR-126-3p-mimic (passenger)**: 5'-G*G*TGCATCGATGCAGG*G*G*G*Gxxxxx rCrArUrUrArUrUrArCrUrCrArCrGrGrUrArCrGrAmArA-3'; **Scramble RNA (scrRNA, guide)** : 5’-rGrGrCrGrUrGrUrArUrUrArArGrGrCrUrArArArUrCrU-3’; **CpG-scrRNA (passenger)** : 5’-G*G*T GCATCGATGCAGG*G*G*G*G xxxxx rArUrUrUrArGrCrCrUrUrArArUrArCrArCrGrCrCmArA-3’, where the asterisk (*) indicates phosphorothioation. One nonbridging atom of oxygen on phosphate was replaced with sulfur. ‘r’ indicates ribo. 'm' indicates the 2′-O-methyl analog of the nucleotide.

***In vitro* treatment of	ECs with TNFα, miR-126 mimics, miR-126 inhibitor (miRisten), TNFα receptor-blocking antibody and TNFα-neutralizing antibody**

Murine BM ECs were treated with murine recombinant (mr) TNFα (0.2, 1, 10ng/ml) (410-MT, R&D Systems) or vehicle, miR-126 mimics or SCR (2µM), miRisten or SCR (1µM), mrTNFα + SCR or mrTNFα + miR-126 mimics, mrTNFα ± anti-TNFαR1 (55R-593, Biolegend) ± anti-TNFαR2 (TR75-32.4, Biolegend) Abs or ± IgG Ab, TNFα-neutralizing Ab (MP6-XT22, Biolegend) or IgG Ab for an indicated period of time; levels of pri/pre-, mature miR-126 and miR-126 target genes *Vcam1* and *Spred1* and frequency of CD31^+^Sca-1^high^ and CD31^+^Sca-1^low^ ECs were assessed.

***In vivo* treatment of normal and AML mice**

To evaluate *in vivo* BM vascular changes induced by mrTNFα and miR-126 level changes, normal wt mice were treated with mrTNFα (1µg/day, ip) versus (vs) vehicle, or with miRisten (20mg/kg/day, iv) vs SCR, for 3 wks. For rescue experiment, normal wt mice were also treated with mrTNFα + miR-126 mimics (20mg/kg/day, iv) or mrTNFα + SCR for 3 wks. To evaluate if EC miR-126 OE could rescue TNFα-induced BM vascular changes, Spred1^flox/flox^/Tie2-cre+ (*Spred1^ECΔ/Δ^*, miR-126 OE in ECs) and Spred1^flox/flox^/Tie2-cre- (wt) mice were treated with mrTNFα (1µg/day, ip) or vehicle for 3 wks.

To determine if TNFα mediated vascular changes occurred during AML cell growth, we performed rescue experiments. *Mll*^PTD/WT^/*Flt3*^ITD/ITD^ AML mice, or recipient mice transplanted with BM MNCs from diseased *Mll*^PTD/WT^/*Flt3*^ITD/ITD^ AML mice, were treated with TNFα-neutralizing Ab (anti-TNFα, 1mg/mouse, ip, 4 times/wk; MP6-XT22, Biolegend) or IgG control Ab for 3 wks.

To evaluate the impact of treatment-induced vascular changes on LSC burden, BM MNCs (CD45.1/CD45.2 or CD45.2, 2x10^6^ cells/mouse) from diseased *Mll*^PTD/WT^/*Flt3*^ITD/ITD^ mice were transplanted by tail vein injection into irradiated (4Gy) congenic recipient mice (CD45.1) to generate a cohort of leukemic mice with similar disease onset time. After confirming AML cell engraftment in PB (40-60%) at 2 wks post transplantation, these mice were treated with AC220 (20mg/kg/day, oral gavage, from Selleckchem) or vehicle for 3 wks. To evaluate the impact of EC miR-126 KO on LSC treatment response, BM MNCs (CD45.1/CD45.2, 2X10^6^ cells/mouse) from diseased *Mll*^PTD/WT^/*Flt3*^ITD/ITD^ mice were transplanted into miR-126^flox/flox^/Tie2-cre+ (miR-126 KO in ECs, also called miR-126*^ECΔ/Δ^*) and miR-126^flox/flox^/Tie2-cre- (wt) mice. After confirming AML cell engraftment in PB (40-60%) at 2 wks post transplantation, these mice were treated with AC220 (20mg/kg/day, oral gavage) for 3 wks.

To evaluate impact of pharmacological EC miR-126 deprivation on LSC burden, recipient mice transplanted with BM MNCs from diseased *Mll*^PTD/WT^/*Flt3*^ITD/ITD^ AML mice were treated with AC220 (20mg/kg/day, oral gavage) ± mrTNFα (1µg/day, ip), or AC220 + miRisten (20mg/kg/day, iv) vs AC220 + SCR, for 3 wks. After 3 wks of treatment, a cohort of AML mice were monitored for leukemia development and survival, and another cohort of mice were euthanized and leukemia (donor AML) and LSC burden (donor AML LSKs) in the BM (right femur) and spleen were analyzed by flow cytometry analysis, and BM vascular changes (tibias) were analyzed by IF staining followed by confocal imaging and flow cytometry analysis of Sca-1^high^ and Sca-1^low^ EC subfractions. BM cells from the left femur of the 2^nd^ cohort of treated mice were pooled and transplanted into 2^nd^ recipient mice (CD45.1, 1×10^6^ cells/mouse), which were monitored for white blood cell (WBC) counts, AML cell engraftment and survival. LSC burden evaluated by the survival of the 2^nd^ recipient mice were compared between the groups.

***In vivo* treatment of AML PDX model**

To study human FLT3-ITD+ AML blasts induced BM vascular changes, CB CD34^+^ cells (1X10^5^/mouse) or AML MNCs (2x10^6^/mouse) were transplanted via tail vein injection into sub-lethally irradiated (3Gy, X-RAD 320 irradiator) 6-10 wks old NSGS mice. Engraftment was monitored every 2 wks by labeling blood cells with anti-mouse CD45 and anti-human CD45 and CD33 antibody followed by flow cytometry analysis. To evaluate impact of pharmacological EC miR-126 deprivation on LSC burden, NSGS mice were transplanted with human FLT3-ITD+ AML blasts. After confirming human CD45^+^ cell engraftment in PB (≥20%) around 4 wks post transplantation, the mice were treated with AC220 (20mg/kg/day, oral gavage) ± mrTNFα (1µg/day, ip), or AC220 + miRisten (20mg/kg/day, iv) vs AC220 + SCR, for 3 wks. After 3 wks of treatment, a cohort of mice were monitored for leukemia development and survival. Another cohort of mice were euthanized, BM (right femur) and spleen cells were analyzed for human CD45^+^ cell engraftment by flow cytometry analysis, and BM vascular changes (tibias) were analyzed by CD31 (FITC) and Sca-1 (PE) IF staining followed by confocal imaging as well as flow cytometry analysis of BM EC Sca-1^high^ and Sca-1^low^ subfractions. BM cells from the left femur of the treated mice were pooled and transplanted into irradiated (3Gy) NSGS recipient mice (1×10^6^/mouse, n=10 mice/group x 4 groups = 40 mice), followed by evaluation of WBC counts, human CD45^+^ cell engraftment and survival. LSC burden evaluated by the survival of the 2^nd^ recipient mice were compared between the groups.

**Reference**

1. Kusumbe, A. P., Ramasamy, S. K., Starsichova, A. & Adams, R. H. Sample preparation for high-resolution 3D confocal imaging of mouse skeletal tissue. Nat Protoc. 2015; 125(10), 1904-1914.

2. Passaro, D. et al. Increased Vascular Permeability in the Bone Marrow Microenvironment Contributes to Disease Progression and Drug Response in Acute Myeloid Leukemia. Cancer Cell. 2017; **32**, 324-341.

3. Kim, D., Paggi, J. M., Park, C., Bennett, C. & Salzberg, S. L. Graph-based genome alignment and genotyping with HISAT2 and HISAT-genotype. Nat Biotechnol. 2019; **37**, 907-915.

4. Liao, Y., Smyth, G. K. & Shi, W. The Subread aligner: fast, accurate and scalable read mapping by seed-and-vote. Nucleic Acids Res. 2013; **41**, e108.

5. Love, M. I., Huber, W. & Anders, S. Moderated estimation of fold change and dispersion for RNA-seq data with DESeq2. Genome Biol. 2014; **15**, 550.

6. Zhang, B. et al. Bone marrow niche trafficking of miR-126 controls the self-renewal of leukemia stem cells in chronic myelogenous leukemia. Nat Med. 2018; **24**, 450-462.
